# Multifaceted analysis of equine cystic echinococcosis: genotyping, immunopathology, and screening of repurposed drugs against *E. equinus* protoscolices

**DOI:** 10.1186/s12917-025-04616-z

**Published:** 2025-03-17

**Authors:** Noha Madbouly Taha, Mai A. Salem, Mohamed A. El-Saied, Faten F. Mohammed, Mohamed Kamel, Mohamed M. El-Bahy, Reem M. Ramadan

**Affiliations:** 1 Department of Parasitology, Faculty of Medicine, Cairo University, Cairo, 11956 Egypt; 2 Department of Parasitology, Faculty of Veterinary Medicine, Cairo University, Giza, 12211 Egypt; 3 Department of Pathology, Faculty of Veterinary Medicine, Cairo University, Giza, 12211 Egypt; 4 Department of Pathology, College of Veterinary Medicine, King Faisal University, Al-Ahsa, 31982 Saudi Arabia; 5 Department of Medicine and Infectious Diseases, Faculty of Veterinary Medicine, Cairo University, Giza, 11221 Egypt

**Keywords:** *Echinococcus equinus*, Genotyping, Gene expression, Immunohistochemistry, Paroxetine, Colmediten, Ator, Brufen, Drug repurposing

## Abstract

Cystic echinococcosis (CE) is a neglected zoonotic disease that causes significant economic losses in livestock and poses health risks to humans, necessitating improved diagnostic and therapeutic strategies. This study investigates CE in donkeys using a multifaceted approach that includes molecular identification, gene expression analysis, serum biochemical profiling, histopathological and immunohistochemical examination, and in vitro drug efficacy evaluation. Molecular analysis of hydatid cyst protoscolices (HC-PSCs) from infected donkey livers and lungs revealed a high similarity to *Echinococcus equinus* (GenBank accession: PP407081). Additionally, gene expression analysis indicated significant increases (*P* < 0.0001) in interleukin 1β (*IL-1β*) and interferon γ (*IFN-γ*) levels in lung and liver homogenates. Serum biochemical analysis showed elevated aspartate transaminase (*AST*), alkaline phosphatase (*ALP*), and globulin levels, alongside decreased albumin compared to non-infected controls. Histopathological examination revealed notable alterations in pulmonary and hepatic tissues associated with hydatid cyst infection. Immunohistochemical analysis showed increased expression of nuclear factor kappa B (*NF-κB*), tumor necrosis factor-α (*TNF-α*), and toll-like receptor-4 (*TLR-4*), indicating a robust inflammatory response. In vitro drug evaluations revealed that Paroxetine (at concentrations of 2.5, and 5 mg/mL) demonstrated the highest efficacy among repurposed drugs against HC-PSCs, resulting in the greatest cell mortality. Colmediten followed closely in effectiveness, whereas both Brufen and Ator exhibited minimal effects. This study identifies Paroxetine as a promising alternative treatment for hydatidosis and provides a framework for investigating other parasitic infections and novel therapies.

## Introduction

Hydatidosis, also known as cystic echinococcosis (CE), is a significant yet neglected zoonotic disease that poses a global public health and economic burden. This parasitic infection originates from the larval stage of the *Echinococcus* species and is mainly transmitted through the consumption of food or water contaminated with parasite eggs. These eggs are excreted in the feces of infected dogs, which serve as the definitive hosts [1]. Humans and various intermediate hosts, including livestock and equines, can become accidentally infected. Once ingested, the parasite develops into cystic lesions, known as hydatid cysts, which predominantly form in the liver and lungs but may also affect other tissues. These cysts, characterized by their slow growth over months or years, can lead to significant inflammation and clinical complications, potentially threatening the host’s health [1].

The complexity of hydatid cysts extends beyond their location and growth patterns. They can be classified into two types based on their contents: fertile cysts, which contain protoscolices (PSCs) capable of developing into adult tapeworms, and sterile cysts, which lack viable PSCs. The structure of the cyst wall reflects the dynamic interaction between the parasite and the host’s immune system. It includes a tough fibrous pericyst, formed as a result of the host’s inflammatory response, followed by a laminated layer produced by the parasite, and an innermost germinal layer, which is responsible for producing PSCs [2]. Understanding this intricate structure is essential for deciphering the parasite’s survival mechanisms within the host and developing targeted therapeutic approaches.

Adding to the complexity of hydatidosis is the genetic diversity within *Echinococcus granulosus* sensu lato (s.l.). Molecular studies based on mitochondrial gene sequences, such as cytochrome C oxidase subunit I (*COX1*) and nicotinamide adenine dinucleotide dehydrogenase subunit I (*ND1*), have identified ten genotypes (G1–G10) within the *E. granulosus* s.l. complex. These genotypes have been reclassified into five distinct species: *E. granulosus* sensu stricto (s.s.) (G1-G3), *E. equinus* (G4), *E. ortleppi* (G5), *E. canadensis* (G6-G10), and *E. felidis* [3, 4]. This genetic variability underscores the need for precise identification of strains to better understand their epidemiology, pathogenicity, and treatment implications.

The intricate relationship between the parasite and host is further demonstrated by the evolving immune response to hydatid cysts over the course of infection. During the early stages, a strong T helper 1 (Th1)-mediated immune response is typically activated, which can eliminate many invading parasites and establish protective immunological memory. However, as the disease progresses, the parasite employs sophisticated immune evasion strategies, leading to a shift toward a T helper 2 (Th2)-dominated response [5]. This Th2 response is characterized by the production of cytokines such as IL-4, IL-13, IL-5, and IL-10, along with the recruitment of eosinophils, alternatively activated macrophages, mast cells, plasma cells, and lymphocytes. This altered immune environment enables the parasite to persist chronically, often with minimal or vague clinical symptoms and a subdued inflammatory response [6–8]. Understanding these immune dynamics is crucial for developing effective therapeutic and preventive strategies.

Given the complex nature of hydatidosis, diagnosing the condition requires a multifaceted approach that combines imaging studies with serological and biochemical evaluations. Biochemical parameters, including liver function tests (aspartate transaminase [AST], alanine transaminase [ALT], alkaline phosphatase [ALP], and creatine kinase [CK]), kidney function tests (creatinine, urea), serum electrolytes (calcium, sodium, potassium, and phosphorus), and serum glucose levels, are routinely used to assess disease progression and the host’s response to treatment [9]. These diagnostic tools provide valuable insights into the systemic effects of hydatid cysts and their impact on organ function.

Once diagnosed, treatment of hydatidosis typically involves a combination of surgical intervention and pharmacotherapy. Surgical options include open or laparoscopic surgery and the percutaneous aspiration-injection-reaspiration (PAIR) technique. While surgery remains a cornerstone of treatment, it carries inherent risks, including high morbidity and mortality, cyst rupture, and the potential for anaphylaxis and recurrence [10]. To complement surgical approaches, Albendazole (ABZ) is widely regarded as the primary pharmacological agent for hydatidosis. ABZ can be used as a standalone treatment or in combination with surgery [11]. However, its therapeutic use requires prolonged high-dose regimens, which are associated with significant side effects, including hepatotoxicity [12, 13]. These limitations highlight the urgent need for alternative treatment options.

The search for new treatments is hampered by the exorbitant costs of drug discovery and development, ranging from $500 million to $2 billion per drug. To address these financial and logistical barriers, drug repositioning or repurposing has emerged as a promising strategy. This approach involves identifying new therapeutic applications for already approved drugs, offering a cost-effective and time-efficient alternative to de novo drug development [14]. Drug repositioning has the potential to accelerate the availability of effective treatments for hydatidosis, thereby improving outcomes for affected populations.

In light of these challenges and opportunities, the current study aims to address critical gaps in understanding and treating hydatidosis by pursuing two key objectives. The first objective is to conduct a comprehensive analysis of hydatid cyst protoscolices (HC-PSCs) from Egyptian donkeys. This analysis will focus on three main aspects: phylogenetic characterization to understand the genetic diversity of the parasite strains; immunological and biochemical profiling of infected donkeys to elucidate host responses; and histopathological and immunohistochemical examination of liver and lung tissues to assess the local impact of infection.

The second objective is to evaluate the in vitro efficacy of four repurposed drugs—atorvastatin, Paroxetine, Colmediten, and ibuprofen—against hydatid PSCs, compared to the current gold-standard anti-hydatid drug, Albendazole. By addressing these objectives, this study seeks to provide novel insights into the pathophysiology of hydatidosis in equines and explore cost-effective treatment strategies, ultimately contributing to the global effort to combat this neglected zoonotic disease.

## Materials and methods

### Samples collection and protoscolices (PSCs) separation

In rural Egypt, donkeys play a crucial role in transporting people and goods, especially in areas without vehicular access. They also support agriculture by moving water, fertilizers, and crops. However, donkeys unable to perform these tasks due to untreated injuries or deformities are often sold for slaughter, where they become food for carnivorous animals in zoos or circuses.

From December 2023 to April 2024, the donkeys were acquired from private individuals who transferred ownership to the Giza National Zoo, where they were intended for use in the zoo’s feeding programs. Selection was not based on infection status since infection could only be confirmed post-mortem. Instead, donkeys were included in the study based on their availability from private sellers and their eligibility for the zoo’s programs. All selected donkeys were over five years old, reflecting a mature population often discarded from labor-intensive tasks due to age-related physical limitations. Upon arrival at the Giza National Zoo, these donkeys underwent initial medical examinations to assess their overall health. The investigated animals underwent multiple diagnostic techniques for various diseases as part of several ongoing studies in our department. Only animals with a confirmed history of hydatid cyst infection were selected for inclusion in this study. Following euthanasia, a thorough post-mortem examination was conducted, during which hydatid cyst infection was diagnosed through palpation and incision. Since infection status was unknown prior to euthanasia, the study represents a random sampling of donkeys entering the zoo, making the findings relevant for understanding the natural prevalence of the disease in this population [15].

Healthy controls were defined as donkeys that showed no visible hydatid cysts upon thorough post-mortem examination, including palpation and incision of the liver and lungs. To rule out infection, macroscopic inspection was performed, and serum biochemical parameters were analyzed to confirm the absence of systemic disease. Additionally, histopathological examination of liver and lung tissues was conducted to ensure the absence of hydatid cysts or inflammatory lesions associated with *Echinococcus* infection. To verify that elevations in inflammatory cytokines were specifically due to hydatidosis, cytokine levels in infected donkeys were compared with those in these rigorously screened healthy controls. Furthermore, no clinical signs or histopathological evidence of concurrent infections were observed, reducing the possibility of confounding factors affecting cytokine levels.

Blood samples were collected at slaughter, and post-mortem inspections included palpation and incision to diagnose and extract any HC present in the carcass and organs. Additionally, representative tissue samples from the lungs and liver were collected from each animal. The collected materials were stored in separate polyethylene bags and promptly transferred to the Parasitology Laboratory at the Faculty of Veterinary Medicine, Cairo University, under strict hygienic conditions and preserved on ice. In the laboratory, tissues surrounding hydatid cysts were carefully removed to prevent rupture. To evaluate the presence of calcification in hydatid cysts, a macroscopic examination was performed to assess their texture, firmness, and structural characteristics. Calcified cysts were identified based on their small size, increased firmness, and gritty consistency upon palpation. To further confirm calcium deposition, Alizarin Red staining was applied, which selectively binds to calcium, allowing for its visualization. Cysts were washed with phosphate-buffered saline (PBS) at pH 7.4, followed by aspiration of hydatid fluid using a sterile needle. The aspirated fluids were examined microscopically, and cysts were identified morphologically according to Manterola et al. 2023 [16]. Fertile cystic fluid containing PSCs was collected from each cyst; the PSCs were allowed to settle by gravity, discarding the upper fluid. The sedimented PSCs were washed three times with PBS at 37 °C [4, 17]. Samples were stored in RPMI-1640 medium (Gibco Laboratories, Grand Island, NY, USA), including media containing active PSCs for genotyping identification sent to the Department of Biotechnology at Cairo University [4].

### Hydatid cyst genotyping and sequencing

Protoscolices were genotyped against reference samples using conventional PCR following the method by Wassermann et al. 2015 [18]. Nucleic acid sequences from 25 positive samples were extracted immediately after collection using a QIAamp DNA Mini Kit (Qiagen, Hilden, Germany), strictly following the manufacturer’s protocol. PCR was performed to identify *Echinococcus equinus* (G4) using specific primers: forward: TTA CTG CTA ATA ATT TTG TGT CAT; reverse: GCA TGA TGC AAA AGG CAA ATA AAC.

The EmeraldAmp GT PCR master mix (Takara, Japan) was used in a reaction volume of 25 µL, incorporating 5 µL of sample DNA as a template and 10 pmol of each primer (Metabion, Germany). The amplification conditions included an initial denaturation step at 94 °C for 3 min, followed by 25 cycles of denaturation at 94 °C for 30 s, annealing at 56 °C for 30 s, extension at 72 °C for 30 s, and a final extension step at 72 °C for 5 min. Specific bands at 1075 bp were confirmed through electrophoresis on a 1.5% agarose gel.

After amplification, PCR products were isolated from agarose gels and purified using the GeneiPureTM Quick PCR Purification Kit (Bangalore Genei). The refined PCR products were sequenced using a Rikakan DNA sequencer (Rikakan, Japan), employing both forward and reverse primers for two COXI mitochondrial genes. DNA sequencing was conducted in both orientations. Nucleotide sequences were analyzed using Finch TV v1.4.0 (Geospira Inc.) to ensure the quality of electropherograms. The extracted sequences were aligned with confirmed *Echinococcus equinus* strains from GenBank using BLAST [19]. Phylogenetic analysis was performed using “Mega 11” software via a neighbor-joining procedure with 1000 bootstrapping replicates. The phylogenetic matrix (phylogenetic identity and phylogenetic distance) is based on the multiple alignment of the PP407081.1 sequence and other related *E. equinus* COXI mitochondrial genes with the Tamura Nei model. The analyzed sequences from this study were deposited in GenBank under accession number PP407081.

### Immuno-expression of cytokines by real-time PCR (RT-PCR)

Total RNA was extracted from lung and liver samples of infected and non-infected donkeys using the RNeasy Mini Kit (Qiagen, Venlo, The Netherlands), following the manufacturer’s instructions. cDNA was synthesized using the QuantiTect Reverse Transcription Kit (Qiagen). The reaction mixture consisted of 10 µL of Sybr Green Master Mix 2X (Bio-Rad), 0.4 µL of primers listed in Table 1 (1 µM) and 1 µL of cDNA template (1 µg), adjusted to a final volume of 20 µL.

Samples were run in duplicate on a 96-well real-time PCR plate (Bio-Rad) using a CFX96 Touch Real-Time PCR machine (Bio-Rad). The cycling profile included an initial step at 50 °C for 2 min, followed by 95 °C for 10 min, then 40 cycles of denaturation at 95 °C for 15 s and annealing/extension at 60 °C for 1 min. Quantification was performed using the comparative ∆∆ cycle threshold method with normalization to glyceraldehyde-3-phosphate dehydrogenase (GAPDH) mRNA levels. Results are expressed as fold changes relative to the healthy control group.

**Table 1:** Oligonucleotide primers utilized in real-time PCR

Equine gene target	Primer sequence	Reference
INF-γ	Forward: TCTTTAACAGCAGCACCAGCAA Reverse: GCGCTGGACCTTCAGATCAT	[20]
IL-1β	Forward: AGTCTTCAGTGCTCAGGTTTCTGA Reverse: TGCCGCTGCAGTAAGTCATC	[21]
GAPDH	Forward: GGCGTGAACCACGAGAAGTATAA Reverse: CCCTCCACGATGCCAAAGT	[22]

### Analysis of biochemical parameters

Blood samples were collected from the jugular veins of the donkeys and immediately transported to the laboratory on ice for analysis. The serum was separated by centrifugation at 3000 g for ten minutes and preserved at − 20 °C until further analysis. Biochemical parameters such as ALP, aspartate aminotransferase (AST), albumin, and globulin levels were measured using commercially available kits [23].

### Histopathological, histochemical and immunohistochemical investigation

Specimens from the lungs and liver of both infected and uninfected donkeys were collected and preserved in 10% neutral buffered formalin. The specimens underwent routine processing using a tissue processor (HistoCore PEARL, Leica, Germany) and were embedded in paraffin blocks before being sectioned at a thickness of 4 μm. Tissue sections received standard Hematoxylin and Eosin (H&E) staining as well as Masson trichrome staining (MTC) following Bancroft and Layton, 2018 [24].

For immunological staining, sections measuring 5 μm in thickness were prepared on adhesive slides, rehydrated, and rinsed in PBS before incubation with primary monoclonal antibodies against TNF-α (sc-52746; Santa Cruz Biotechnology), TLR4 antibody (sc-293072; Santa Cruz Biotechnology), and nuclear factor-κB (NF-κB) (sc-8008; Santa Cruz Biotechnology), diluted at 1:200 overnight at 4 °C in a humid chamber. Endogenous peroxidase activity was blocked using hydrogen peroxide before applying an HRP-labeled detection kit (Bio SB, USA) according to the manufacturer’s instructions for developing positive reactions.

### Drug repurposing against HC-PSCs in vitro

#### Tested drugs and their doses

For drug repurposing against HC-PSCs in vitro, four drugs were evaluated for their efficacy against PSCs. Atorvastatin was administered orally as Ator (Egyptian International Pharmaceutical Industries Co.) at a dosage of 20 mg/70 kg body weight and tested in solution at concentrations of 20 mg/mL and 40 mg/mL. Ibuprofen was provided as Brufen 400 mg tablets by Abbott Drug Company and administered orally at doses of 4–10 mg/kg body weight; it was tested at concentrations of 20 mg/mL and 40 mg/mL. Paroxetine was supplied as film-coated tablets by EVA Pharma Company with a daily oral dose not exceeding 50 mg; it was tested at concentrations of 1.25 mg/mL, 2.5 mg/mL, and 5 mg/mL. Colchicine was provided as Colmediten tablets by Kahira Pharmaceuticals and tested at concentrations of 100 µg/mL and 200 µg/mL. Albendazole (ABZ) was used as a drug control at a concentration of 100 µg/mL [25], provided in suspension form by Pharma Cure Pharmaceuticals Company, Egypt. All drugs were diluted to the desired concentrations in RPMI-1640 medium [26].

#### Experimental design

Fresh PSCs were harvested from fertile hydatid cysts containing clear cystic fluid and examined microscopically before being directly transferred into RPMI media maintained at 37 °C for drug exposure studies. Drug concentrations were adjusted within warm RPMI-1640 medium in test tubes before being added to sterile Petri dishes containing approximately 10^3^ viable PSCs per mL (viability > 90%) [27]. A PSC suspension in a sterile RPMI medium served as the negative control.

Three replicates per drug concentration were prepared simultaneously, with dishes incubated at 37 °C during exposure periods ranging from 6 to 72 h. Following each exposure time point, media containing drugs were discarded; precipitated PSCs underwent three washes with warm RPMI-1640 medium free from drugs before re-incubation for an additional 48 h at 37 °C.

To evaluate drug efficacy, the viability of settled PSCs was assessed based on contractility and developmental stage. A methylene blue exclusion test using a solution of methylene blue at a concentration of 0.1% differentiated between living and dead PSCs. Drug efficacy was calculated according to Salama et al. 2012 [28]:

Mortality % = (Mean Survived PSCs in Control - Mean Survival in Exposed) / Mean Survival in Control × 100.

Additionally, lethal concentrations causing death in 50% (LC50) and 100% (LC100) of PSCs were determined following methodologies outlined by Farhadi et al. 2021 [29].

### Statistical analysis

Statistical analyses were conducted using both SPSS and GraphPad Prism software. An unpaired t-test was employed for the biochemical and gene expression data, with significance defined as a P-value < 0.001 [30, 31]. For LD50 evaluation, a nonlinear regression model was utilized. Additionally, a one-way ANOVA followed by multiple comparison Fisher’s LSD test was applied to compare differences between groups [32, 33].

## Results

### Prevalence and genetic characterization of *Echinococcus equinus*

**Fig. 1:**
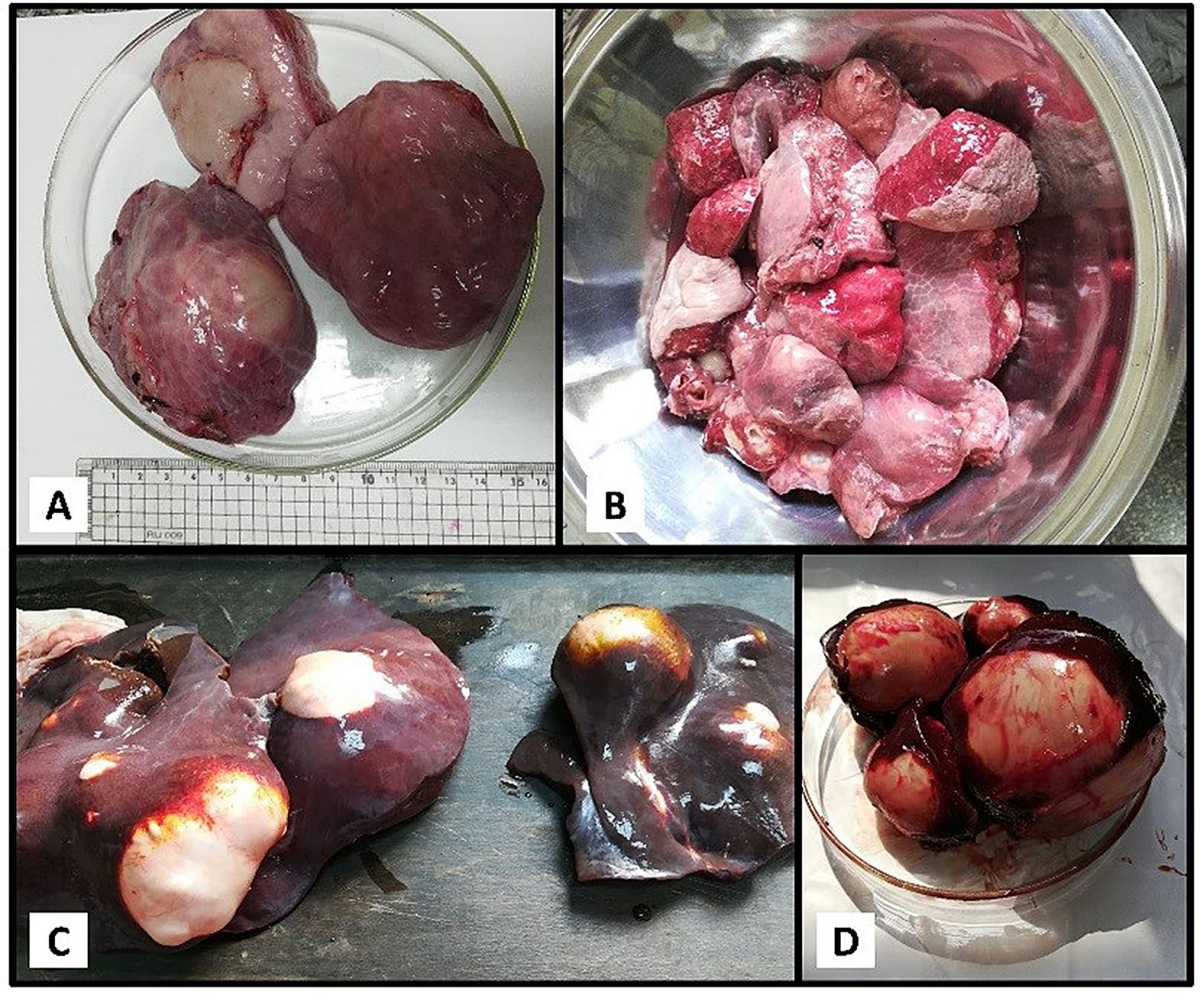
**A** and **B**. Freshly collected hydatid cyst in the lung of slaughtered donkeys (≥ 5 cm); **C** and **D**. Hydatid cyst found in the liver of slaughtered donkeys (2–4 cm)

Of the 65 donkeys examined at Egypt’s Giza National Zoo, 25 (38.46%) were infected with HC. These cysts were predominantly found in both the lungs and liver of the infected animals (Fig. 1). Most cysts (> 25) varied in size from 2 to 7 cm in diameter and contained viable protoscolices (PSCs), indicating fertility. However, three cysts were sterile (lacking PSCs), and four were small and calcified.

**Fig. 2:**
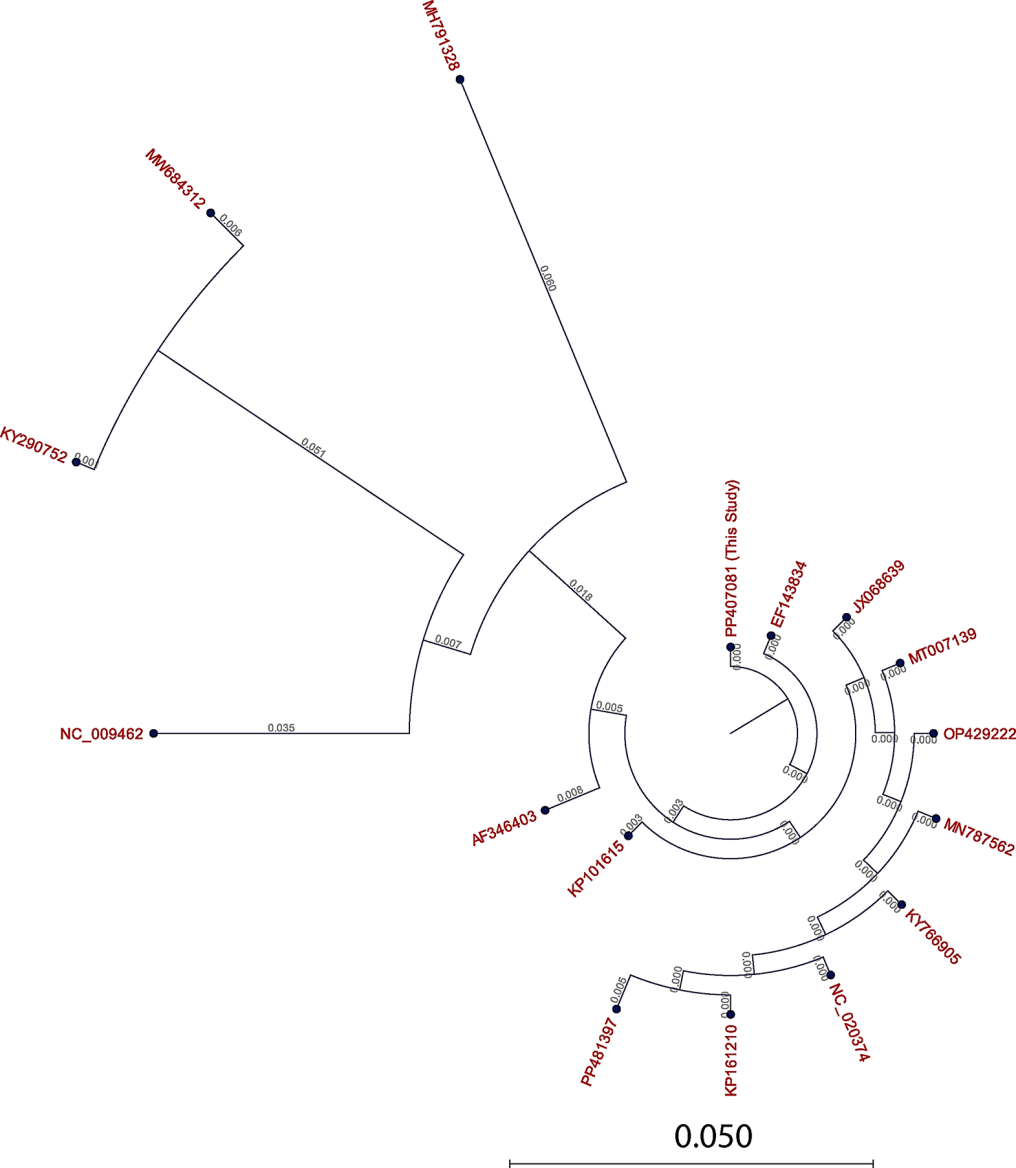
The phylogenetic tree created using the neighbor-joining method and *E. equinus* COXI mitochondrial genes, including the new sequence with accession number PP407081 and other related sequences downloaded from GenBank

**Fig. 3:**
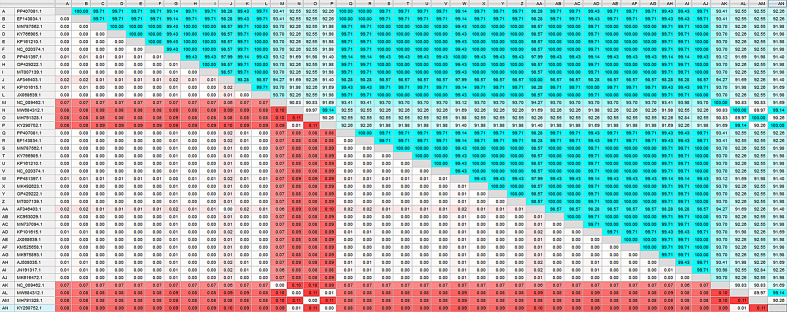
The phylogenetic matrix (phylogenetic identity in the upper part and phylogenetic distance in the lower part) is based on the multiple alignments of the PP407081.1 sequence and other related *E. equinus* COXI mitochondrial genes

To further characterize the parasite, we conducted a genetic analysis. The cytochrome c oxidase subunit I (*COXI*) sequence from the isolate (GenBank accession: PP407081) revealed high homology to *E. equinus*. The phylogenetic analysis tree and matrix table displaying genetic identity and distance are shown in Figs. 2 and 3. The phylogenetic analysis revealed 100% similarity to the reference sequence EF143834, with a genetic distance of 0.000. It showed 99.77% similarity to sequences MN787562, PP504258, and KY766905, and 99.43% homology with KP101615.1 (see Figs. 2 and 3). This genetic characterization not only confirms the presence of *E. equinus* in Egyptian donkeys but also provides crucial epidemiological data for future control strategies.

### Immunological and biochemical profiles

**Fig. 4:**
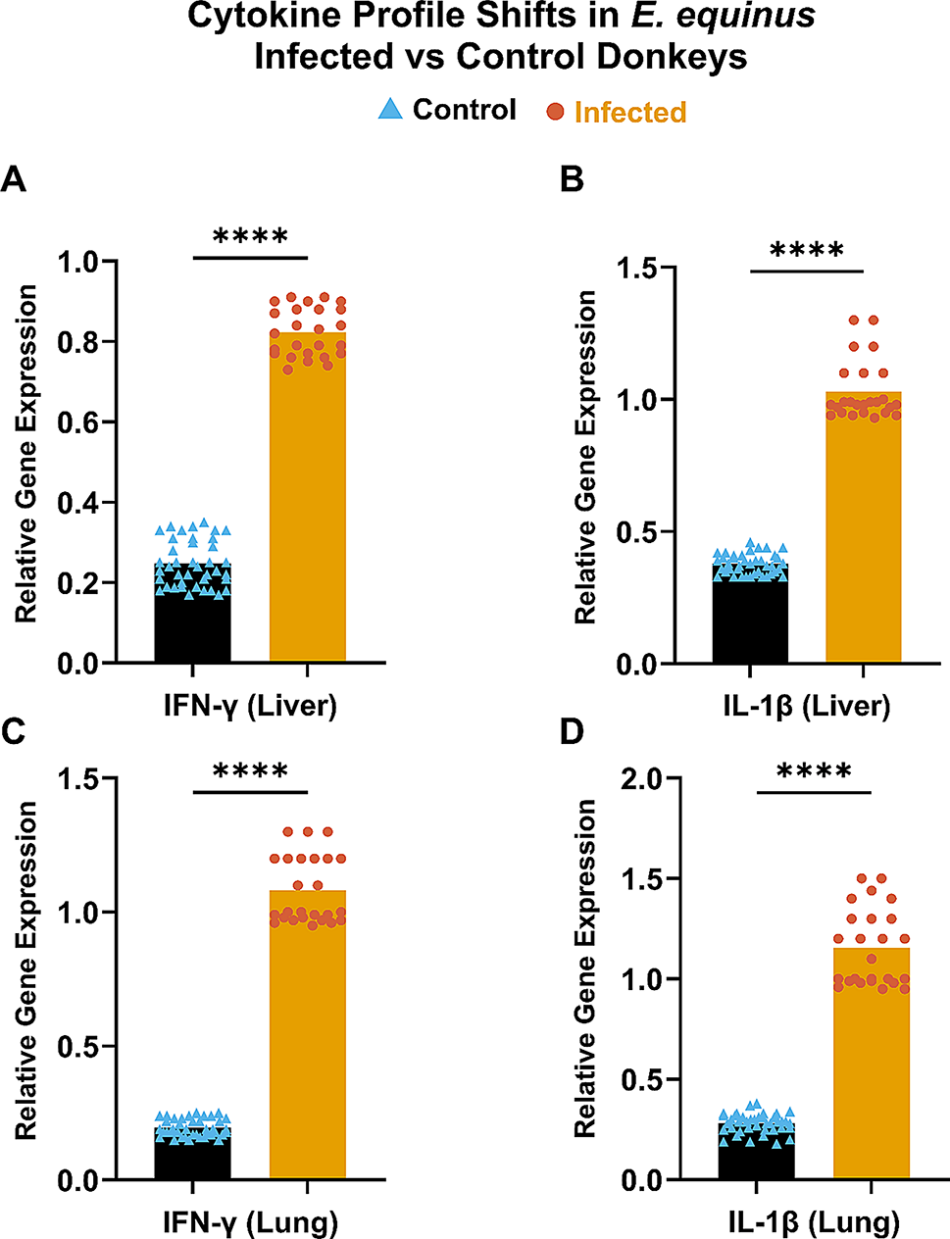
Gene expression of different cytokines in liver and lung tissues infected with HC. This figure illustrates the differences in key cytokines parameters between healthy control donkeys and those affected by *E. equinus*: (**A**) Interferon gamma (IFN-γ) in the liver, (**B**) Interleukin 1 beta (IL-1β) in the liver (**C**) IFN-γ in the lung, and (**D**) IL-1β in the lung. Statistically significant differences between the control and CE-infested groups were determined using a two-tailed T-test and are marked by asterisks (****P < 0.0001)

Building on our genetic findings, we next investigated the impact of HC infection on host physiology through cytokine gene expression and serum biochemistry. We observed significant elevations in *IFN-γ* and *IL-1β* mRNA levels in both liver and lung tissues of infected animals compared to healthy controls (*P* < 0.0001) (Fig. 4). Specifically, *IFN-γ* mRNA levels showed a 3.33-fold increase in the liver and a 5.54-fold increase in the lung, while *IL-1β* mRNA levels exhibited a 2.73-fold increase in the liver and a 4.10-fold increase in the lung. This substantial increase in pro-inflammatory cytokines indicates a robust immune response to the parasite.

**Fig. 5:**
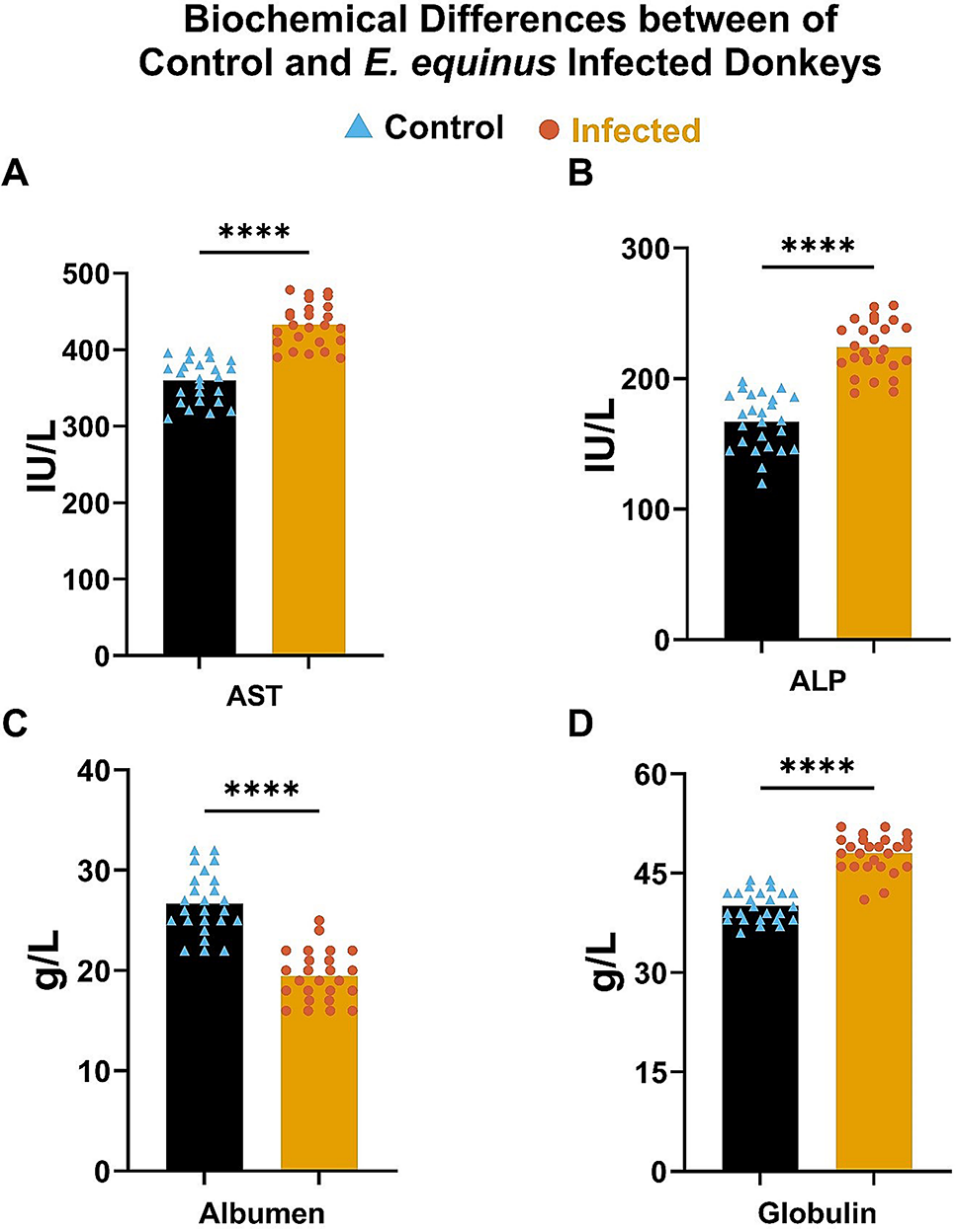
Biochemical Parameter Levels in Healthy Control Donkeys and Donkeys Infested with *E. equinus*. This figure illustrates the differences in key biochemical parameters between healthy control donkeys and those affected by *E. equinus*: (**A**) aspartate aminotransferase (AST), (**B**) alkaline phosphatase (ALP), (**C**) albumin, and (**D**) globulin. ****P < 0.0001 indicates significant differences between the groups

Concurrently, serum biochemical analysis revealed marked alterations in liver function parameters. Infected donkeys showed significantly increased ALP and AST enzyme activities and higher globulin levels, while albumin levels decreased significantly compared to healthy controls (*P* < 0.0001) (Fig. 5). These changes reflect the systemic impact of hydatidosis on host metabolism and liver function, underscoring the complex host-parasite interaction.

### Histopathological and immunohistochemical findings

**Fig. 6:**
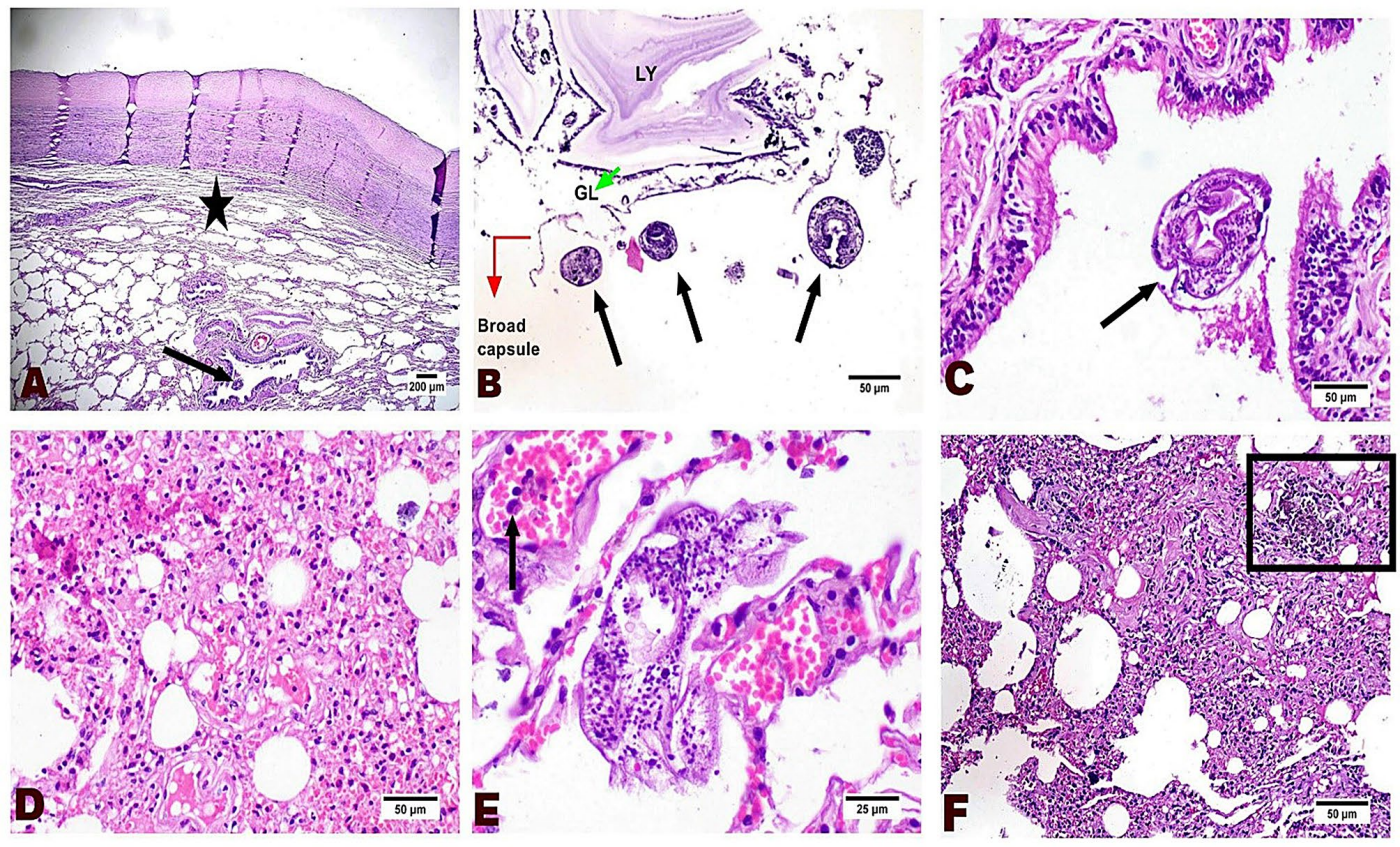
Photomicrograph of pulmonary tissue reveals a hydatid cyst with a thick adventitial layer compressing the underlying alveoli (star), along with parasitic stages present in the bronchiolar lumen (arrow) (**A**). The cyst contains many scolices (arrows) within the brood capsule, featuring a thin germinal layer (GL) and a thick laminated layer (LY) (**B**). The bronchiolar wall shows hyperplastic proliferation of epithelium with parasites in their lumina (**C**). The alveolar wall exhibits necrosis of the alveolar epithelium, edema, capillary congestion, and inflammatory cell infiltration (**D**). The alveolar epithelium shows necrosis with the attachment of a parasite scolex to the alveolar wall, accompanied by capillary congestion and inflammatory cell extravasation (arrow) (**E**). Alveolar fibroplasia is observed, with the formation of a hyaline membrane associated with mononuclear cell infiltration (**F**) and hemosiderin pigment deposition (insert box)

To further elucidate the effects of hydatid infection at the tissue level, we conducted a microscopic examination of infected tissues. This analysis revealed distinct characteristics of hydatid cysts in the lungs and liver. Pulmonary cysts exhibited numerous scolices in brood capsules, thin germinal layers, and thick laminated and adventitial layers with minimal inflammation, indicating fertility (Fig. 6A-B). The surrounding lung tissue showed alveolar collapse, bronchiolar epithelial hyperplasia, vascular congestion, edema, and inflammatory cell infiltration (Fig. 6C-F).

**Fig. 7:**
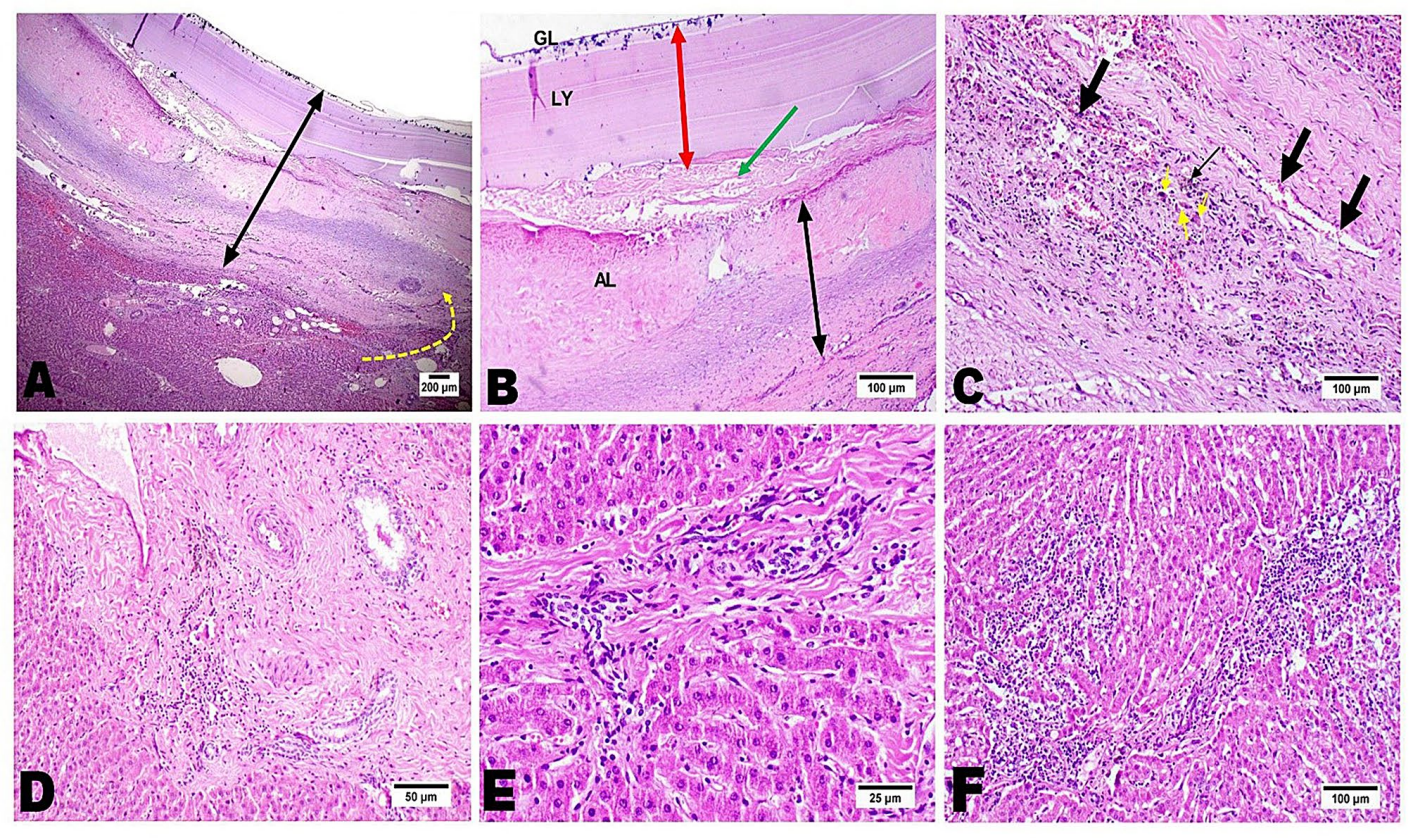
Photomicrograph of hepatic tissue showing the presence of hydatid cyst with a marked thick wall (double arrowhead) with the interconnection of the adventitial layer of the parasitic wall into the hepatic bridging fibrosis (yellow arrow) and marked hepatic hemorrhage underneath the cyst wall (**A**). The cyst wall consists of thin germinal layer (GL) with no noticed scolices, thick laminated layer (red double head arrow, LY), and an extremely hyalinized collagenous adventitial wall (AL, double black head arrow) (**B**) The collagenous adventitial layer replacing the host tissue and showed marked angiogenesis (black thick arrows) with mononuclear cells infiltration that laden with hemosiderin pigment (black thin arrows) with extensive necrosis of hepatocytes (yellow arrows) (**C**). Severe portal fibrosis and ductular reaction with lymphocytic infiltration (**D**). Bridging fibrosis with oval cell proliferation in interlobular areas (**E**). Marked inflammatory reaction comprising the hepatic parenchyma (**F**)

In contrast, hepatic lesions were more pronounced, with cysts displaying thicker laminated and adventitial layers interconnected with dense collagen fibers (Fig. 7A-C). The liver parenchyma exhibited portal fibrosis, ductular reaction, steatosis, and hemosiderin-laden macrophages (Fig. 7D-F).

**Fig. 8:**
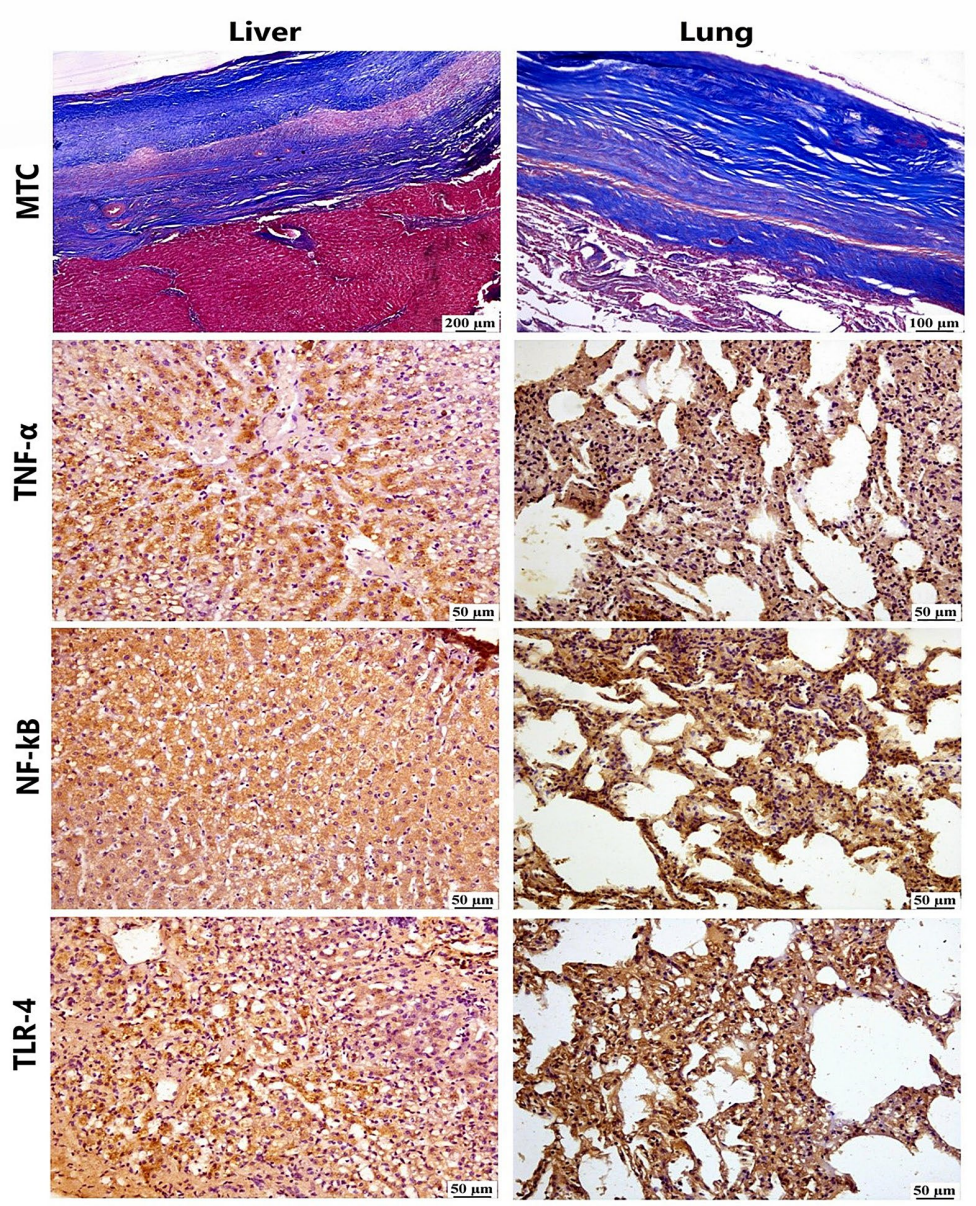
Photomicrograph of liver and lung tissues, intense blue staining of the adventitial layer of cyst wall with MTC in liver and lung tissues. There is positive TNF-α expression in the Kupffer cells and hepatocellular cytoplasm, with moderate expression in the cytoplasm of macrophages infiltrating the alveolar wall. There is marked nuclear NF-κB translocation in hepatocytes and alveolar macrophages. The immunophenotyping of TLR-4 is observed in HSCs, hepatocellular cytoplasm and alveolar macrophages

To gain deeper insights into the inflammatory response, we performed immunohistochemical analysis. This revealed increased expression of inflammatory markers. TNF-α expression was elevated in both pulmonary and hepatic tissues, with higher intensity in the liver. NF-κB showed marked nuclear translocation in hepatocytes, while TLR-4 expression was detected in the alveolar epithelium, hepatocytes, and inflammatory cells (Fig. 8). These findings underscore the complex and tissue-specific inflammatory response to hydatid infection.

### Efficacy of repurposed drugs

#### Comparative effectiveness of paroxetine, colmedetin, brufen, and ator on *E. equinus*: an analysis of dose and concentration

**Fig. 9:**
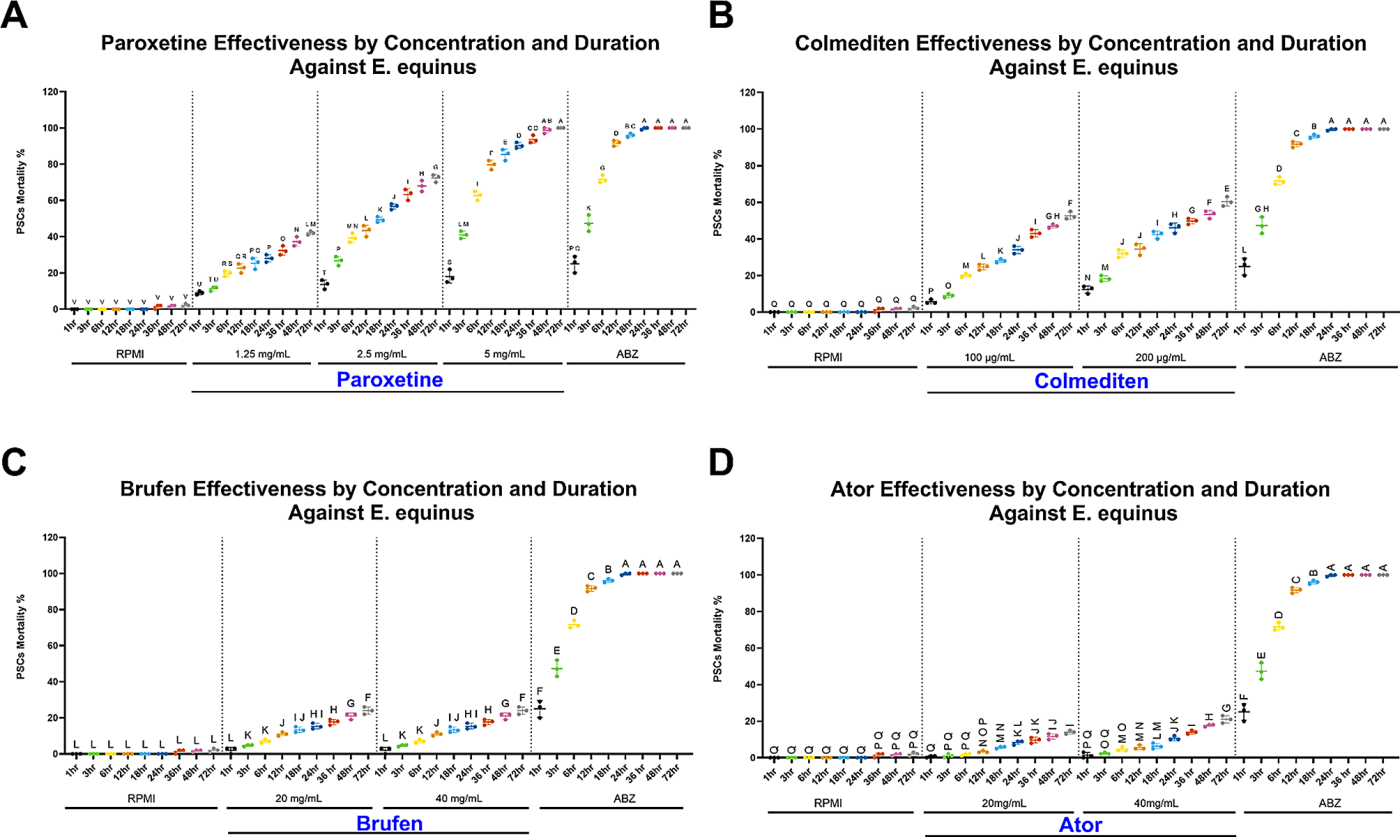
Dose-dependent and duration effectiveness of compounds against *E. equinus*. (**A**) Paroxetine effectiveness at concentrations of 2.5 mg/mL and 5 mg/mL. (**B**) Colmediten effectiveness at concentrations of 100 µg/mL and 200 µg/mL. (**C**) Brufen effectiveness at concentrations of 20 mg/mL and 40 mg/mL. (**D**) Ator effectiveness at concentrations of 20 mg/mL and 40 mg/mL. RPMI was used as the negative control, and ABZ served as the positive control. The mortality percentage was calculated using the formula: Mortality Percentage = [(Number of Non-Viable PSCs) / (Total Number of PSCs)] × 100. Different letters indicate statistically significant differences (*p* < 0.05)

The efficacy of four pharmacological agents—Paroxetine, Colmedetin, Brufen, and Ator—was evaluated against *E. equinus* by assessing the percent mortality of PSCs across varying concentrations and time points. Distinct patterns in the time and concentration-dependent effectiveness of these drugs are noted in Fig. 9. Paroxetine demonstrated a progressive increase in PSC mortality with rising concentrations (1.25 mg/mL, 2.5 mg/mL, and 5 mg/mL) and extended exposure durations. At a concentration of 1.25 mg/mL, significant yet minimal mortality was observed at early time points (1 to 6 h), with substantial increases after 24 h, reaching peak efficacy at 72 h. Higher concentrations (5 mg/mL) showed accelerated effects, with notable mortality as early as 12 h.

Additionally, the mortality rate of PSCs caused by Colmedetin increased significantly with higher concentrations, particularly noticeable at 6 and 12 h, reaching optimal levels beyond 24 h. The 200 µg/mL concentration consistently outperformed the 100 µg/mL across all time points.

Brufen, evaluated at concentrations of 20 mg/mL and 40 mg/mL, showed minimal cytotoxic effects on PSCs. The 40 mg/mL concentration exhibited a more significant mortality rate than 20 mg/mL. While both concentrations showed time-dependent increases, the higher concentration achieved more efficacy, indicating a dose-dependent response. However, its effect was still not great.

Ator, similar to Brufen, was assessed at 20 mg/mL and 40 mg/mL. Ator’s mortality impact was significant from 12 h onwards, with the 40 mg/mL concentration yielding superior effects compared to the 20 mg/mL. The most pronounced effect was observed at the 72-hour mark for both concentrations but was still low compared to ABZ, Paroxetine, and Colmedetin.

**Fig. 10:**
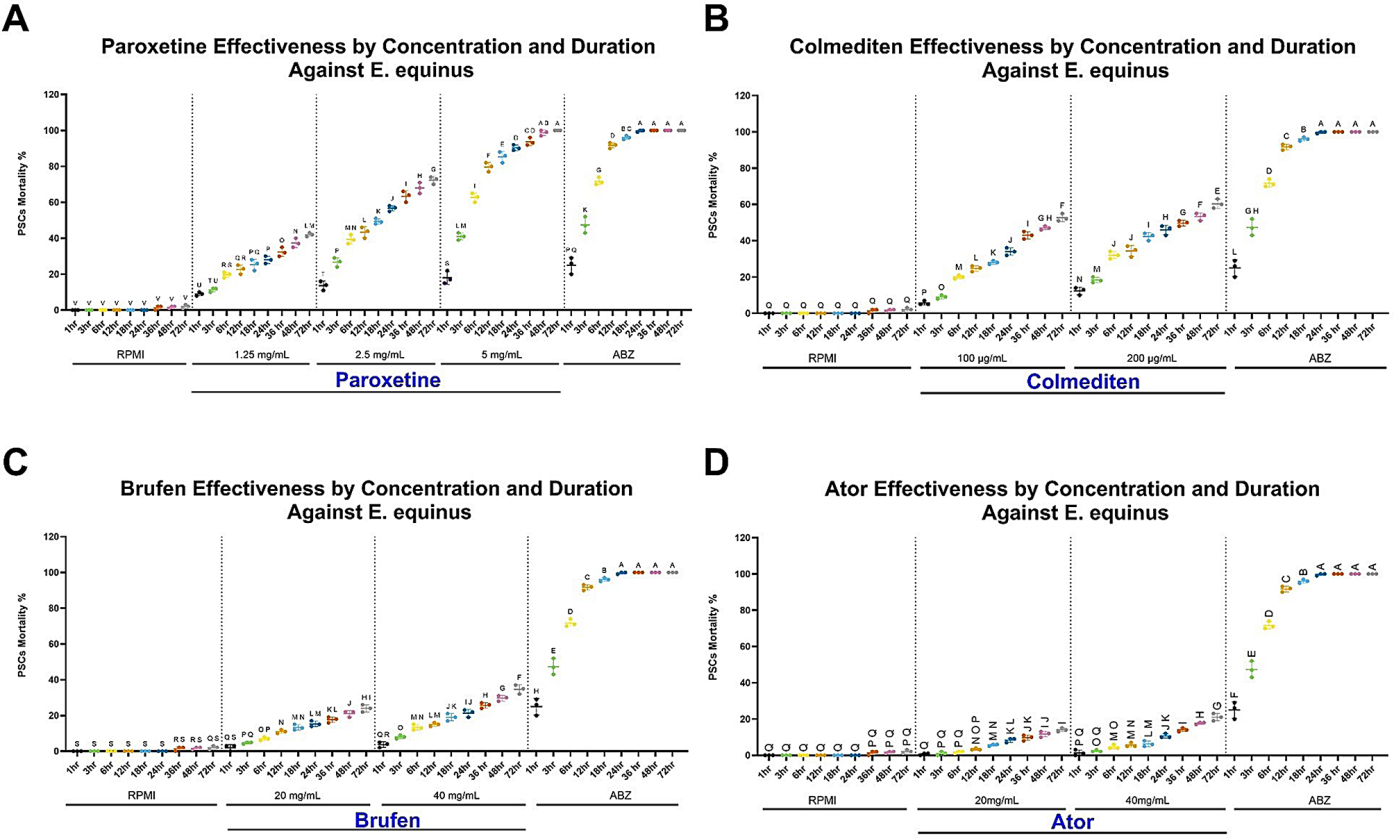
Comparative effectiveness of Paroxetine, Colmedetin, Brufen, and Ator against *Echinococcus equinus* as measured by percent mortality of PSCs. The graph presents data across varying concentrations (1.25 mg/mL, 2.5 mg/mL, 5 mg/mL for Paroxetine; 100 µg/mL, 200 µg/mL for Colmedetin; 20 mg/mL, 40 mg/mL for Brufen and Ator) and multiple time points (1 h, 3 h, 6 h, 12 h, 18 h, 24 h, 36 h, 48 h, and 72 h)

Our data (Fig. 10) indicate a significant variation in the effectiveness of the four drugs against *E. equinus*. Paroxetine demonstrated the highest mortality rates at lower concentrations over time, particularly at 5 mg/mL, achieving nearly 100% mortality at 72 h. Colmedetin also showed promising results, particularly at higher concentrations (200 µg/mL), with mortality rates exceeding 90% after 48 h. Brufen exhibited moderate effectiveness with a gradual increase in mortality that plateaued around 40% at its highest concentration (40 mg/mL) after 72 h. Ator showed the least efficacy among the tested drugs, with maximum mortality rates reaching only about 50% at the highest concentration after 72 h.

#### Lethal dose 50% (LD50%)

**Fig. 11:**
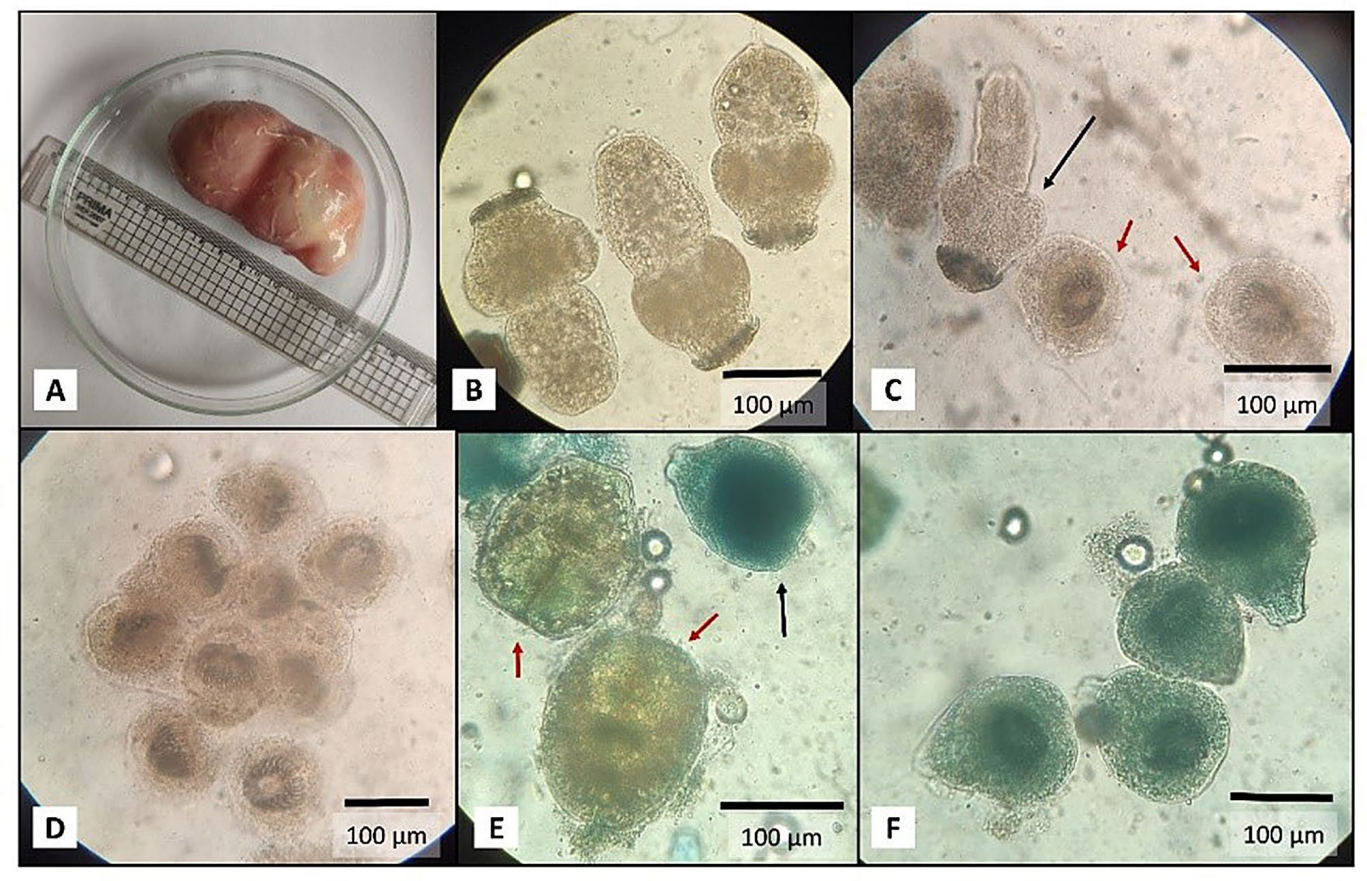
**(A)** Hydatid cyst freshly collected from slaughtered Equine. **(B)** Viable *E. equinus* PSCs after 72 h of incubation showing maturation (suckers development and primitive strobili) and refractile granules. **(C)** Viable (Black arrow) and dead (Red arrows) PSCs. **(D)** Dead PSCs showing no maturation. **(E)** Unstained viable (Red arrows) and stained dead (Black arrow) PSCs using MB exclusion test. **(F)** Stained dead PSCs using MB exclusion test

**Fig. 12:**
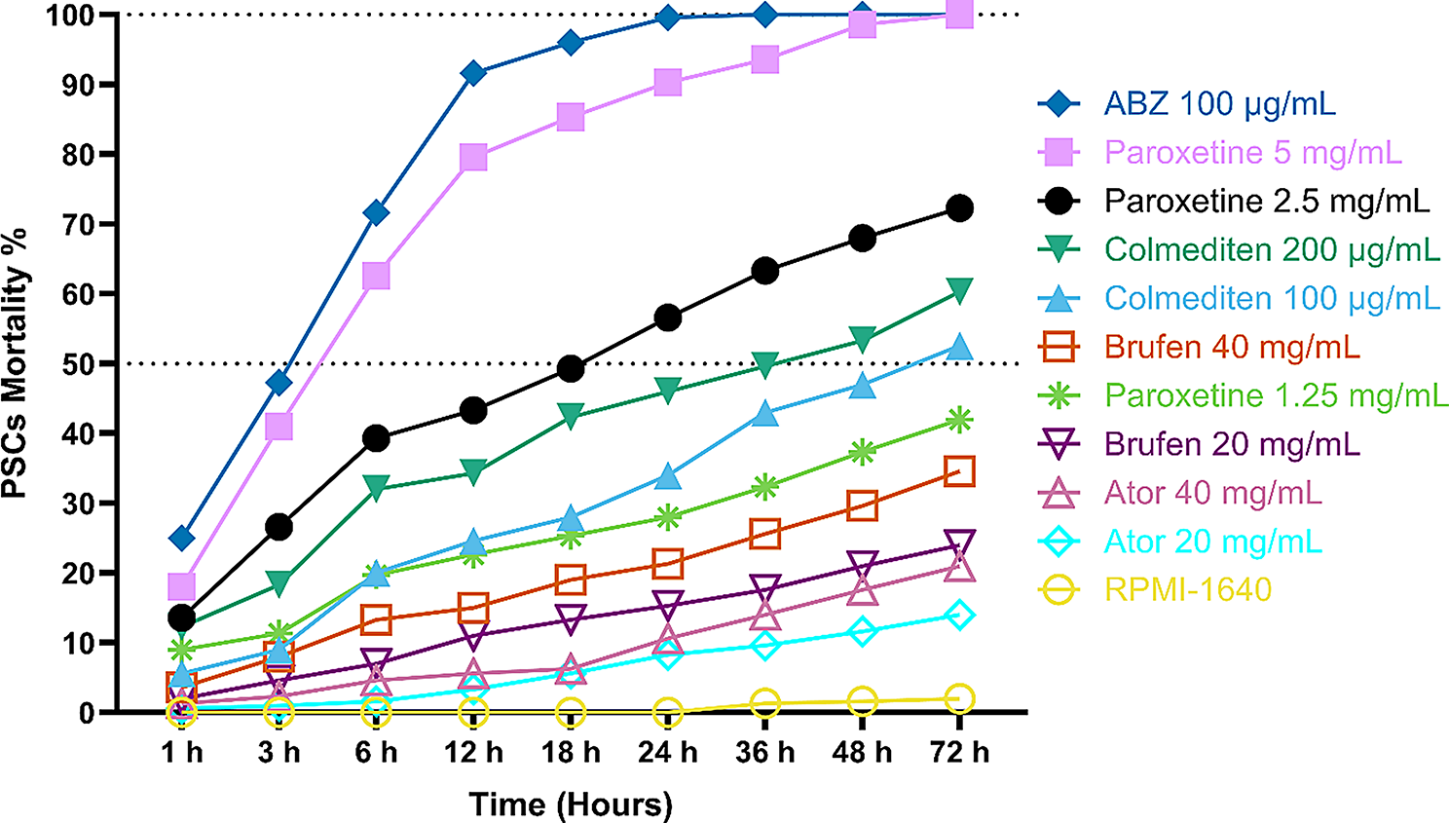
Determination of LD50 and LD100 for Paroxetine (1.25, 2.5 and 5 mg/mL), Colmediten (100 and 200 µg/mL), Brufen (20 and 40 mg/mL), Ator (20 and 40 mg/mL), Albendazole (ABZ, 100 µg/mL), with RPMI serving as the negative control

**Fig. 13:**
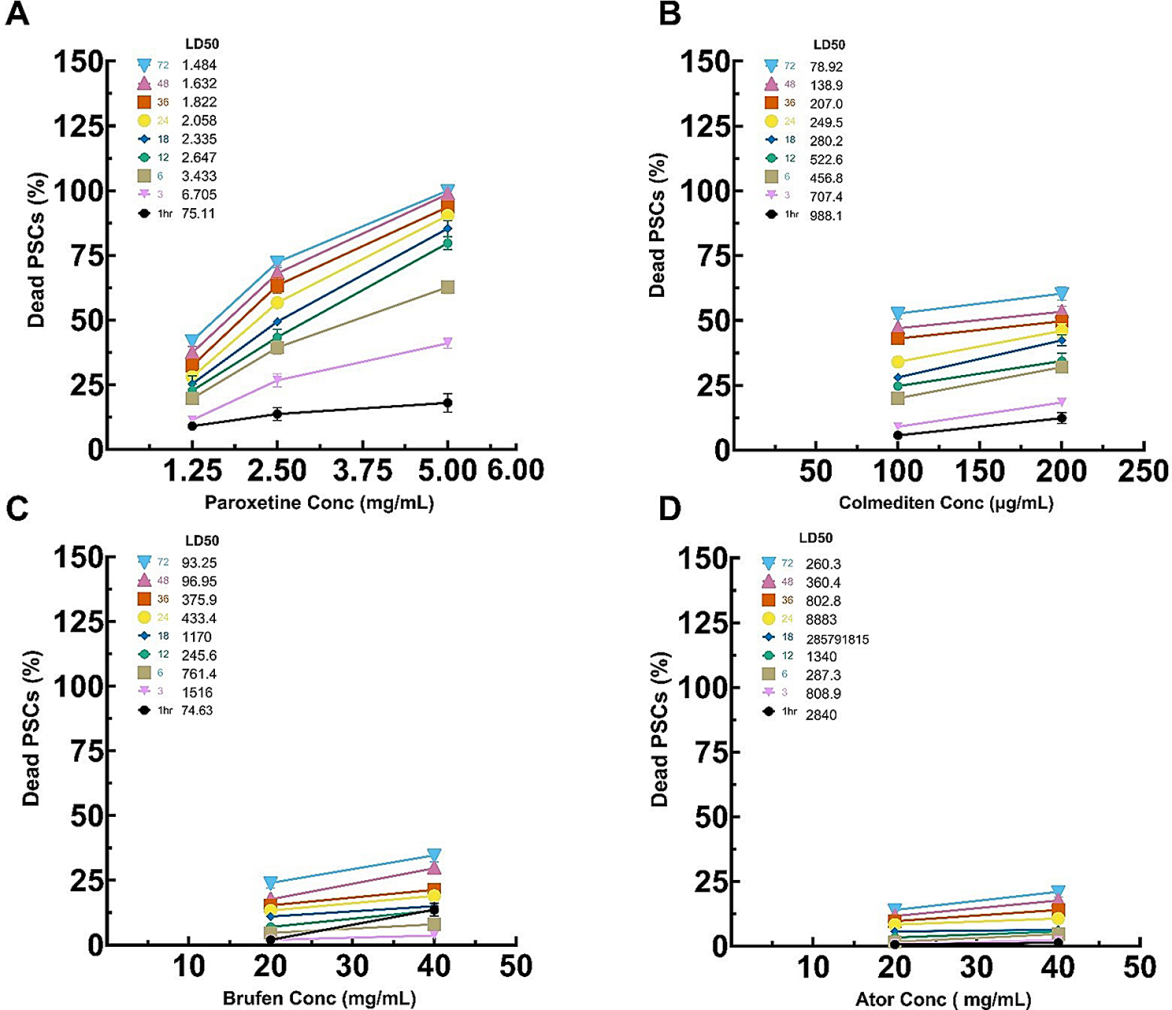
Further Determination of LD50 for Paroxetine (2.5 and 5 mg/mL), Colmediten (100 and 200 µg/mL), Brufen (20 and 40 mg/mL), Ator (20 and 40 mg/mL), Albendazole (ABZ, 100 µg/mL), with RPMI serving as the negative control based on non-linear regression model in different time points

Given the significant pathological effects of hydatid infection, we next explored potential therapeutic interventions through drug repurposing. In vitro testing of repurposed drugs against *E. equinus* PSCs yielded varying results. Brufen and Atorvastatin (Ator) showed poor efficacy, with maximum mortality rates of 34.6% and 21%, respectively, after 72 h at the highest concentrations tested (Figs. 9, 10, 11, 12 and 13).

Colchicine (Colmediten) demonstrated moderate efficacy, with LC50 reached at 100 µg/mL after 72 h and at 200 µg/mL after 48 h. Notably, Paroxetine emerged as the most promising candidate, showing potent scolicidal activity. Its LC50 was observed at 2.5 mg/mL after 24 h and at 5 mg/mL after 6 h, with 100% mortality achieved at 5 mg/mL after 72 h (Table 2; Figs. 9, 10, 11, 12 and 13). Expanded LD50 for all four drugs was demonstrated in all the different time points in Fig. (12).

For comparison, we tested the reference drug, Albendazole (ABZ), which showed the highest efficacy, achieving 100% PSCs mortality after 36 h. Importantly, there was no significant difference between the efficacy of Paroxetine at 5 mg/mL and ABZ, suggesting Paroxetine’s potential as an alternative treatment for hydatidosis.

These results highlight the potential of drug repurposing in developing new treatments for hydatidosis, with Paroxetine emerging as a promising candidate for further investigation. The varying efficacies observed among the tested drugs underscore the importance of targeted screening in the search for novel anti-parasitic agents.

**Table 2:** In vitro LC50 and LC100 values of repurposed drugs against *E. equinus* PSCs recorded LC_50_ and LC_100_ of the repurposed drugs against *E. equinus* PSCs in vitro

Investigated Drug	LC_50_	LC_100_
Colmediten 100 µg/mL	100 µg/mL / 72 h	--
Colmediten 200 µg/mL	200 µg/mL / 48 h	--
Paroxetin 2.5 mg/mL	2.5 mg/mL / 24 h	--
Paroxetin 5 mg/mL	5 mg/mL / < 6 h	5 mg/ml / 72 h
ABZ 100 µg/mL	100 µg/mL / < 6 h	100 µg/ml / 36 h

## Discussion

Hydatidosis, also known as CE, is a significant zoonotic disease with severe implications for human and animal health worldwide [4]. The disease is characterized by the development of large cysts, ranging in size from a hen’s egg to a child’s head, which can form in various organs, causing pressure on surrounding tissues and carrying the risk of rupture and subsequent anaphylaxis [34]. Understanding the epidemiology and genetic characteristics of the parasite is crucial for developing effective control strategies.

### Genetic characterization and epidemiology

In equine hosts, infection occurs when animals graze on pastures contaminated with dog and fox feces containing parasite eggs. Donkeys and horses serve as intermediate hosts, developing viable hydatid cysts with protoscolices primarily in their lungs and liver. In Egypt, infected donkeys, particularly when their carcasses are improperly disposed of near canals or agricultural lands, become a significant source of infection for the primary hosts of *Echinococcus equinus*, namely dogs and foxes [3].

Our genetic characterization of hydatid cyst protoscolices revealed that the cytochrome oxidase subunit I sequence was closely related to *E. equinus*. The sequence was deposited in GenBank under accession number PP407081, showing 100% homology with the sequence reported by Varcasia et al. 2008 in Italy [35], 99.77% homology with those reported by Kinkar et al. 2017, Alvarez Rojas et al. 2020, and Wang et al. 2023 in Estonia, Switzerland, and China, respectively [36–38], and 99.44% homology with that reported by Aboelhadid et al. 2013 in Egypt [3]. This genetic identification of the echinococcosis strain in Egyptian donkeys provides valuable information for disease control strategies and sets the stage for understanding the host-parasite interactions at the molecular level.

### Immunological and biochemical profile

Building on the genetic characterization, our study aimed to further elucidate the immunological cytokine and cellular inflammatory profile in the lungs and liver of chronic equine CE and their relationship with cyst fertility. We observed significant increases in IFN-γ and IL-1β mRNA levels in both lungs and liver with fertile and sterile hydatid cysts, characteristic of the inflammatory response against the metacestode. IL-1β is widely produced by WBCs in CE-infected intermediate hosts, especially close to the metacestode, and is assumed to play a crucial immune-modulatory function in ensuring parasite persistence [19]. IFN-γ inhibits the development and function of parasitic helminths by stimulating macrophages to produce nitric oxide (NO) [39]. These findings align with observations from previous studies [20, 40–42]. However, our results differ from those reported by [6, 39], who observed no variations in gene expression between normal and infected animals.

Complementing the immunological findings, our biochemical analysis revealed significant elevations in serum AST and ALP activities in infected donkeys compared to healthy controls, consistent with previous findings in donkeys infected with *E. equinus* [43]. This elevation in enzyme activities may be explained by damage to liver cells triggered by hypoxia associated with anemia. We also observed increased globulin levels and decreased albumin levels in infected animals. The increased globulin fraction is attributed to chronic inflammation and responses to antigenic stimulation in the hepatic tissue, while decreased albumin levels may be due to acute phase reactions and hepatic dysfunction [44]. These biochemical changes further underscore the systemic impact of hydatidosis and highlight the need for effective treatment strategies.

### Current treatment approaches and limitations

Given the complex host-parasite interactions and systemic effects of hydatidosis, current treatment approaches face significant challenges. Surgical interventions and PAIR technique are associated with complications such as cyst rupture, anaphylaxis, recurrence, and toxicity from scolicidal agents [13]. Albendazole (ABZ) remains the primary pharmacological treatment but requires high doses for extended periods, leading to serious side effects, including hepatotoxicity [12, 45]. The need for safer, effective anti-hydatidosis drugs is evident, but the high cost of developing new anti-helminthic compounds, estimated at $500 million to $2 billion per drug, presents a significant challenge [14, 46]. These limitations in current treatment options motivated our exploration of drug repurposing as a potential solution.

## Evaluation of repurposed drugs

In response to the challenges associated with current treatments, our study evaluated the efficacy of four repurposed drugs (Atorvastatin, Paroxetine, Colchicine, and Ibuprofen) against hydatid protoscolices in vitro, compared to ABZ. Albendazole (ABZ) is widely regarded as the primary pharmacological treatment for different parasites, as it disrupts parasite microtubule formation, leading to impaired glucose uptake and eventual parasite death [47]. We utilized extracted live PSCs derived from fresh uncalcified cysts through aspiration.

Paroxetine, a selective serotonin reuptake inhibitor (SSRI), is widely used for managing anxiety disorders and other conditions like diabetic neuropathy, chronic headaches, and menopausal symptoms [48]. It also shows potential as an antiparasitic agent. Our study demonstrated Paroxetine’s efficacy with an LC50 at 2.5 mg/mL after 24 h and at 5 mg/mL in less than 6 h, reaching an LC100 at 5 mg/mL after 72 h. This aligns with its known effectiveness against parasites such as *Caenorhabditis elegans*, *Trichuris muris*, *Ancylostoma caninum*, and *Schistosoma mansoni* [49].

These effects are linked to Paroxetine’s influence on the serotonergic system, which is crucial for the nervous system of *Echinococcus multilocularis*, impacting proliferation, vesicle integrity, and parasite stem cell viability [50, 51]. Serotonin transport is highly essential for *Echinococcus* vesicle development and parasite growth in the liver, aiding metacestode re-differentiation. Disrupting this pathway has been shown to reduce metacestode viability and compromise parasite stem cell integrity. Previous research has demonstrated that serotonin modulation can impair the proliferation of *E. multilocularis* vesicles, significantly affecting parasite survival [50]. Similar effects have been reported in other helminths, such as *C. elegans*, *T. muris*, and *A. caninum*, where SSRIs interfere with neuromuscular function, leading to reduced motility and viability [49]. These findings align with our results, suggesting that Paroxetine may exert its anti-parasitic effects through serotonin transporter inhibition, thereby disrupting key developmental processes in *Echinococcus equinus*. 

Colchicine (Colmediten) demonstrated notable anti-protoscolicidal efficacy, achieving LC50 values at concentrations of 100 µg/mL and 200 µg/mL after 72 and 48 h, respectively. This efficacy is attributed to colchicine’s ability to inhibit tubulin polymerization, a mechanism shared with some anti-helminthic drugs [52, 53]. Colchicine and related compounds target microtubules, which are essential for parasite cytoskeleton integrity, cellular division, and motility. The efficacy of tubulin-targeting drugs in helminthic infections has been well established, with benzimidazole derivatives such as Albendazole acting through a similar mechanism [52]. The competitive inhibition of tubulin polymerization by Colmediten is comparable to the mode of action of mebendazole, which binds to helminth β-tubulin and disrupts microtubule formation [54]. Further supporting colchicine’s potential in anti-helminthic therapy, Köhler and Bachmann (1981) showed that mebendazole competitively inhibits colchicine’s attachment to tubulin in *Ascaris suum*, highlighting tubulin as a target for helminth chemotherapy [53]. Additionally, Ranjan et al. (2017) identified key pharmacophore points in the colchicine binding domains of helminth β-tubulin in *Haemonchus contortus*, which serve as effective targets for anti-helminthic drugs. Recent studies on colchicine-based *de novo* prodrugs revealed significant efficacy against *C. elegans* after 22 h of incubation, suggesting promising potential for developing effective anti-helminthic compounds [54]. 

Ibuprofen (Brufen) exhibited poor protoscolicidal efficacy, with a mortality of 34.6% at the highest used dose after 72 h of incubation. However, it is worth noting that ibuprofen has shown protective effects against chicken coccidiosis, both alone and when combined with clopidol. These effects are attributed to ibuprofen’s ability to block COX2 activity through nitric oxide inhibition, decreasing prostaglandins and augmenting IL-2 secretion [55]. Furthermore, ibuprofen has demonstrated potential synergistic therapeutic efficacy when combined with vitamin E and selenium in managing coccidiosis in broilers [56]. Atorvastatin (Ator) also showed poor protoscolicidal efficacy, with 21% mortality at the highest used dose after 72 h of incubation. Despite these results, atorvastatin and other statins have shown efficacy against various parasites in previous studies. These include dose-dependent effects against *S. mansoni*[57], reduction of inflammatory reactions in cerebral malaria models [58, 59], inhibition of *Toxoplasma* growth and invasion [60, 61], efficacy against *Leishmania* [62, 63], and synergistic effects with Nitazoxanide against *Cryptosporidium* in immunosuppressed animals [14]. These outcomes are attributed to statins’ ability to inhibit the mevalonate pathway, thereby decreasing isoprenoid production, which not only improves endothelial function and reduces inflammation through mechanisms like COX2 nitrosylation but also enhances their potential as therapeutic agents beyond cholesterol-lowering, particularly when combined with other treatments [60, 61, 63, 64].

ABZ showed significant dominance over all repurposed drugs except Paroxetine (5 mg/mL), with slight or no significant difference recorded between ABZ and Paroxetine in mortality percentage. Previous studies indicate that ABZ is highly effective against hydatid cysts, with about 70% mortality after one hour at 20 mg/cm³ [65] and 99% mortality after two hours at 10 µg/mL [25].

Our comprehensive analysis of the genetic, immunological, and biochemical aspects of hydatidosis in equine hosts, coupled with the evaluation of repurposed drugs, provides valuable insights into this complex disease. While Paroxetine and Colchicine showed promising results, further in vivo studies are warranted to validate their efficacy and safety as potential treatments for hydatidosis. This research contributes to the ongoing efforts to develop more effective and safer therapeutic options for both human and veterinary medicine in the fight against this globally neglected zoonotic disease. A more intensive investigation into the efficacy of the tested drugs is required using an experimental animal model to evaluate their impact on the general health condition, ensuring a comprehensive assessment of their therapeutic potential and safety profile. This will form the basis for advancing research through preclinical and clinical investigations. Additionally, more detailed mechanistic studies are warranted to further elucidate Paroxetine’s mode of action, which remains an important area for future research, while comprehensive pharmacokinetic and pharmacodynamic studies specific to CE treatment will also be essential in this process. The use of Paroxetine in combination with existing therapeutic approaches, such as the PAIR (puncture, aspiration, injection, re-aspiration) technique, will require collaborative efforts with tropical medicine specialists and careful consideration of patient safety. These efforts will be critical to validating the efficacy and safety of Paroxetine in living systems, ultimately ensuring its suitability as a potential therapeutic option for CE. As part of a future outlook, combining Paroxetine with albendazole (ABZ), a widely used anti-parasitic drug, represents an exciting avenue for further exploration. The synergistic potential of this combination could enhance therapeutic efficacy, particularly in resistant or difficult-to-treat cases of CE. This combined approach could also address the limitations of monotherapy and improve treatment outcomes, warranting focused studies to evaluate their efficacy and safety in both preclinical and clinical settings.

## Conclusion

Our comprehensive analysis of hydatid cyst protoscolices (HC-PSCs) from Egyptian donkeys has yielded significant insights into the genetic, immunological, and biochemical aspects of hydatidosis, as well as potential alternative treatments. The BLAST analysis of the cytochrome c oxidase subunit I (*COX1*) sequence extracted from HC-PSCs revealed a close relation to *Echinococcus equinus*, with the sequence deposited in GenBank under accession number PP407081. This genetic foundation provides two key advantages: (i) it contributes valuable information to the understanding of the epidemiology of hydatidosis in equine hosts in Egypt, and (ii) it establishes a genetic reference for future studies aiming to track and control the disease in this region.

Moving from genetics to physiological impacts, infection by hydatid cysts was found to cause significant alterations in the biochemical profile of infected animals, as evidenced by changes in serum parameters. Furthermore, we observed increased gene expression of inflammatory cytokines in lung and liver homogenates, reflecting the host’s immune response to the parasite. These findings highlight three important insights: (i) the significant physiological burden hydatid cysts impose on equine hosts, (ii) the critical role of inflammatory cytokines in shaping the host-parasite interactions, and (iii) the potential for these biochemical and immunological parameters to serve as diagnostic or therapeutic targets in chronic hydatidosis. Taken together, these data bridge the gap between genetic and clinical manifestations of the disease and lay the groundwork for more targeted interventions.

In light of these insights, we turned our attention to potential treatments. In our evaluation of repurposed drugs as potential anti-helminthic treatments, Paroxetine at 5 mg/mL demonstrated promising efficacy against hydatid disease, with no significant difference observed between its effects and those of the currently used drug, Albendazole (ABZ). This result suggests two crucial advantages: (i) Paroxetine could serve as a viable alternative treatment for hydatidosis, potentially offering improved safety or cost-effectiveness, and (ii) it expands the therapeutic landscape for hydatid disease, particularly in cases where ABZ may not be suitable. Similarly, Colchicine (Colmediten) also showed potential as a protoscolicidal agent, warranting further investigation. In contrast, Ibuprofen (Brufen) and Atorvastatin (Ator) exhibited poor scolicidal efficacy under the tested conditions, underscoring the importance of targeted drug selection and the specificity of anti-helminthic effects among repurposed drugs.

Expanding on the significance of our drug repurposing approach, it is noteworthy that this study represents the first investigation of these repurposed drugs against *E. equinus* genotype 4 of hydatid cysts in Egypt. This novel approach offers two critical advantages: (i) it opens new avenues for treatment development for hydatidosis, and (ii) it potentially reduces the time and cost associated with bringing new therapies to market. These findings underscore the value of drug repurposing in addressing neglected zoonotic diseases and provide a promising path forward in the fight against hydatidosis.

While our findings are promising, it is important to acknowledge several limitations and future directions. First, in vivo studies are needed to confirm the efficacy and safety of Paroxetine and Colchicine as treatments for hydatidosis, bridging the gap between in vitro results and clinical application. Second, the poor efficacy of Ibuprofen and Atorvastatin in our study does not preclude their potential effectiveness at different doses or in combination therapies, which could be explored in future research. Lastly, the genetic and immunological data gathered in this study could inform the development of more targeted therapies or diagnostic tools for hydatidosis in equine hosts, paving the way for personalized treatment approaches.

Collectively, this study provides a multi-faceted approach to understanding and addressing hydatidosis in Egyptian donkeys by combining genetic characterization, immunological profiling, and drug repurposing strategies. Our findings contribute to the growing body of knowledge on this neglected zoonotic disease and offer potential new avenues for its treatment. As we continue to face the challenges posed by parasitic infections worldwide, such integrated approaches will be crucial in developing effective, safe, and accessible interventions for both human and veterinary medicine. By bridging the gaps between genetic insights, immunological responses, and therapeutic interventions, we move closer to comprehensive solutions for combating hydatidosis and similar parasitic diseases.

## Data Availability

(1) Data is provided within the manuscript. (2) Sequence deposited in GenBank under accession number: PP407081.

## References

[1] Hajizadeh M, Jabbari A, Spotin A, Hejazian SS, Mikaeili Galeh T, Hassannia H, et al. Modulatory effects of hydatid cyst fluid on a mouse model of experimental autoimmune encephalomyelitis. Vet Sci. 2024;11:34. 10.3390/vetsci11010034. 38250940 10.3390/vetsci11010034 PMC10819194

[2] Bhalla VP, Paul S, Klar E. Hydatid disease of the liver. Visc Med. 2023;39:112–20. 10.1159/000533807. 37899792 10.1159/000533807 PMC10601525

[3] Aboelhadid SM, El-Dakhly KM, Yanai T, Fukushi H, Hassanin KM. Molecular characterization of Echinococcus granulosus in Egyptian donkeys. Vet Parasitol. 2013;193:292–6. 10.1016/j.vetpar.2012.11.019. 23246076 10.1016/j.vetpar.2012.11.019

[4] Ramadan RM, Khalifa MM, El-Akkad DM, Abdel-Wahab AM, El-Bahy MM. Animal hydatid cyst genotypes as a potential substitute for human hydatid cyst as a source of antigen for diagnosis of zoonotic hydatidosis. J Parasit Dis. 2021;45:424–34. 10.1007/s12639-020-01309-2. 34295041 10.1007/s12639-020-01309-2 PMC8254676

[5] Tamarozzi F, Mariconti M, Neumayr A, Brunetti E. The intermediate host immune response in cystic echinococcosis. Parasite Immunol. 2016;38:170–81. 10.1111/pim.12301. 26683283 10.1111/pim.12301

[6] Siracusano A, Delunardo F, Teggi A, Ortona E. Cystic echinococcosis: aspects of immune response, Immunopathogenesis and immune evasion from the human host. Endocrine’ Metabolic Immune Disorders-Drug Targets. 2012;12:16–23. 10.2174/187153012799279117. 22214328 10.2174/187153012799279117

[7] Díaz Á. Immunology of cystic echinococcosis (hydatid disease). Br Med Bull. 2017;124:121–33. 10.1093/bmb/ldx033. 29253150 10.1093/bmb/ldx033

[8] Siracusano A, Delunardo F, Teggi A, Ortona E. Host-parasite relationship in cystic echinococcosis: an evolving story. Clin Dev Immunol. 2012;2012:639362. 10.1155/2012/639362. 22110535 10.1155/2012/639362 PMC3206507

[9] Özdek U, Oğuz B, Kömüroğlu AU, Değer Y. Determination of the levels of serum oxidative indicator, cytokine and some biochemical parameters in horses naturally infected with theileria equi. Ankara Üniversitesi Veteriner Fakültesi Dergisi. 2020;67:257–63. 10.33988/auvfd.603305.

[10] Akhan O. Percutaneous treatment of liver hydatid cysts: to PAIR or not to PAIR. Curr Opin Infect Dis. 2023;36:308–17. 10.1097/qco.0000000000000956. 37548385 10.1097/QCO.0000000000000956

[11] Dehkordi AB, Sanei B, Yousefi M, Sharafi SM, Safarnezhad F, Jafari R, Darani HY. Albendazole and treatment of hydatid cyst, review of literature. Infect Disorders - Drug Targets. 2018. 10.2174/1871526518666180629134511. 10.2174/1871526518666180629134511 29956639

[12] Taha NM, Youssef FS, Auda HM, El-Bahy MM, Ramadan RM. Efficacy of silver nanoparticles against Trichinella spiralis in mice and the role of multivitamin in alleviating its toxicity. Sci Rep. 2024;14(1):5843. 38462650 10.1038/s41598-024-56337-2 PMC10925591

[13] Alvi MA, Khan S, Ali RMA, Qamar W, Saqib M, Faridi NY, et al. Herbal medicines against hydatid disease: A systematic review (2000–2021). Life (Basel). 2022;12:676. 10.3390/life12050676. 10.3390/life12050676 PMC9145516 35629345

[14] Madbouly Taha N, Salah A, Yousof H-A, El-Sayed SH, Younis AI, Ismail Negm MS. Atorvastatin repurposing for the treatment of cryptosporidiosis in experimentally immunosuppressed mice. Exp Parasitol. 2017;181:57–69. 10.1016/j.exppara.2017.07.010. 28764965 10.1016/j.exppara.2017.07.010

[15] Simsek S, Roinioti E, Eroksuz H. First report of Echinococcus equinus in a Donkey in Turkey. Korean J Parasitol. 2015;53:731–5. 10.3347/kjp.2015.53.6.731. 26797441 10.3347/kjp.2015.53.6.731 PMC4725237

[16] Manterola C, Rivadeneira J, Pogue SD, Rojas C. Morphology of Echinococcus granulosus Protoscolex. Int J Morphology. 2023;41:646–53. 10.4067/s0717-95022023000200646.

[17] El Akkad DMH, Ramadan RM, Auda HM, Abd El-Hafez YN, El-Bahy M, Abdel-Radi S. Improved Dot-ELISA assay using purified sheep coenurus cerebralis antigenic fractions for the diagnosis of zoonotic coenurosis. World’s Veterinary J. 2022;237–49. 10.54203/scil.2022.wvj30.

[18] Wassermann M, Aschenborn O, Aschenborn J, Mackenstedt U, Romig T. A sylvatic lifecycle of Echinococcus equinus in the Etosha National park, Namibia. Int J Parasitol Parasites Wildl. 2014;4:97–103. 10.1016/j.ijppaw.2014.12.002. 25830103 10.1016/j.ijppaw.2014.12.002 PMC4356735

[19] Ramadan RM, Mahdy OA, El-Saied MA, Mohammed FF, Salem MA. Novel insights into immune stress markers associated with myxosporeans gill infection in nile tilapia (molecular and immunohistochemical studies). PLoS ONE. 2024;19(6):e0303702. 10.1371/journal.pone.0303702. 38833454 10.1371/journal.pone.0303702 PMC11149867

[20] Ramadan RM, Bakr AF, Fouad E, Mohammed FF, Abdel-Wahab AM, Abdel-Maogood SZ, El-Bahy MM, Salem MA. Novel insights into antioxidant status, gene expression, and immunohistochemistry in an animal model infected with camel-derived trypanosoma evansi and theileria annulata. Parasites Vectors. 2024;17(1):474. 10.1186/s13071-024-06564-3. 39558410 10.1186/s13071-024-06564-3 PMC11575088

[21] Ramadan RM, Taha NM, Auda HM, Elsamman EM, El-Bahy MM, Salem MA. Molecular and immunological studies on theileria equi and its vector in Egypt. Exp Appl Acarol. 2024;93(2):439–58. 10.1007/s10493-024-00933-4. 38967736 10.1007/s10493-024-00933-4 PMC11269342

[22] Salem MA, Taha NM, El-Bahy MM, Ramadan RM. Phylogenetic position of the pigeon mite, ornithonyssus Sylviarum, with amplification of its Immunogenetic biomarkers in Egypt. Sci Rep. 2024;14(1):22026. 10.1038/s41598-024-72433-9. 39322649 10.1038/s41598-024-72433-9 PMC11424627

[23] Taha NM, Sabry MA, El-Bahy MM, Ramadan RM. Awareness of parasitic zoonotic diseases among pet owners in Cairo, Egypt. Veterinary Parasitology: Reg Stud Rep. 2024;51:101025. 10.1016/j.vprsr.2024.101025. 10.1016/j.vprsr.2024.101025 38772640

[24] Suvarna KS, Layton C, Bancroft JD. Bancroft’s theory and practice of histological techniques. Elsevier health sciences; 2018.

[25] Hosseini MJ, Youssefi MR, Abouhosseini M. Comparison of the effect of Artemisia sieberi essential oil and albendazole drug on protoscolices of hydatid cyst under in vitro conditions. J Babol Univ Med Sci. 2017;19:63–8.

[26] El-Bahy MM, Kamel NO, Auda HM, Ramadan RM. A smart economic way to control camel parasites and improve camel production in Egypt. Exp Parasitol. 2023;255:108650. 10.1016/j.exppara.2023.108650. 37914150 10.1016/j.exppara.2023.108650

[27] Cheraghipour K, Beiranvand M, Zivdari M, Amiri S, Masoori L, Nourmohammadi M, et al. In vitro potential effect of Pipper longum methanolic extract against protoscolices of hydatid cysts. Exp Parasitol. 2021;221:108051. 10.1016/j.exppara.2020.108051. 33301754 10.1016/j.exppara.2020.108051

[28] Salama MM, Taher EE, El. Bahy MM. Molluscicidal and mosquitocidal activities of the essential oils of Thymus capitatus L. and Marrubium vulgare L. Am J Drug Discovery Dev. 2012;2:204–11. 10.3923/ajdd.2012.204.211. 10.1590/s0036-46652012000500008 22983292

[29] Farhadi M, Haniloo A, Rostamizadeh K, Ahmadi N. In vitro evaluation of albendazole-loaded nanostructured lipid carriers on Echinococcus granulosus microcysts and their prophylactic efficacy on experimental secondary hydatidosis. Parasitol Res. 2021;120:4049–60. 10.1007/s00436-021-07343-0. 34669034 10.1007/s00436-021-07343-0

[30] Chan YH. Biostatistics 103: qualitative data - tests of independence. Singap Med J. 2003;44:498–503. 15024452

[31] Taha NM, Zalat RS, Khaled E, Elmansory BM. Evaluation of the therapeutic efficacy of some essential oils in experimentally immunosuppressed mice infected with Cryptosporidium parvum. J Parasit Dis. 2023;47:733–43. 10.1007/s12639-023-01621-7. 38009149 10.1007/s12639-023-01621-7 PMC10667177

[32] Ramadan RM, Salem MA, Mohamed HI, Orabi A, El-Bahy MM, Taha NM. Dermanyssus gallinae as a pathogen vector: phylogenetic analysis and associated health risks in pigeons. Veterinary Parasitology: Reg Stud Rep. 2025;57:101198. 10.1016/j.vprsr.2025.101198. 10.1016/j.vprsr.2025.101198 39855842

[33] Salem MA, Mahdy OA, Ramadan RM. Ultra-structure, genetic characterization and immunological approach of fish borne zoonotic trematodes (Family: Heterophyidae) of a redbelly tilapia. Res Vet Sci. 2024;166:105097. 38007971 10.1016/j.rvsc.2023.105097

[34] Sadr S, Lotfalizadeh N, Abbasi AM, Soleymani N, Hajjafari A, Roohbaksh Amooli Moghadam E, Borji H. Challenges and prospective of enhancing hydatid cyst chemotherapy by nanotechnology and the future of nanobiosensors for diagnosis. Trop Med Infect Dis. 2023;8:494. 10.3390/tropicalmed8110494. 37999613 10.3390/tropicalmed8110494 PMC10674171

[35] Varcasia A, Garippa G, Pipia AP, Scala A, Brianti E, Giannetto S, et al. Cystic echinococcosis in equids in Italy. Parasitol Res. 2008;102:815–8. 10.1007/s00436-007-0862-7. 18180956 10.1007/s00436-007-0862-7

[36] Kinkar L, Laurimäe T, Sharbatkhori M, Mirhendi H, Kia EB, Ponce-Gordo F, et al. New mitogenome and nuclear evidence on the phylogeny and taxonomy of the highly zoonotic tapeworm Echinococcus granulosus sensu stricto. Infect Genet Evol. 2017;52:52–8. 10.1016/j.meegid.2017.04.023. 28456662 10.1016/j.meegid.2017.04.023

[37] Alvarez Rojas CA, Kronenberg PA, Aitbaev S, Omorov RA, Abdykerimov KK, Paternoster G, et al. Genetic diversity of Echinococcus multilocularis and Echinococcus granulosus sensu Lato in Kyrgyzstan: the A2 haplotype of E. multilocularis is the predominant variant infecting humans. PLoS Negl Trop Dis. 2020;14:e0008242–0008242. 10.1371/journal.pntd.0008242. 32401754 10.1371/journal.pntd.0008242 PMC7219741

[38] Wang Y, Zhang J, Wang X, Ahmed H, Shen Y, Cao J. Molecular epidemiology and the control and prevention of cystic echinococcosis in China: what is known from current research. Zoonoses. 2023. 10.15212/zoonoses-2023-0009.

[39] de Biase D, Prisco F, Pepe P, Bosco A, Piegari G, d’Aquino I, et al. Evaluation of the local immune response to hydatid cysts in sheep liver. Vet Sci. 2023;10:315. 10.3390/vetsci10050315. 37235398 10.3390/vetsci10050315 PMC10220960

[40] Mondragón-De-La-Peña C, Ramos-Solís S, Barbosa-Cisneros O, Rodríguez-Padilla C, Tavizón-García P, Herrera-Esparza R. *Echinococcus granulosus*down regulates the hepatic expression of inflammatory cytokines IL-6 and TNFα in BALB/c mice. Parasite. 2002;9:351–6. 10.1051/parasite/2002094351. 10.1051/parasite/2002094351 12514950

[41] Amri M, Aissa SA, Belguendouz H, Mezioug D, Touil-Boukoffa C. *In Vitro*Antihydatic action of IFN-γIs dependent on the nitric oxide pathway. J Interferon Cytokine Res. 2007;27:781–8. 10.1089/jir.2007.0003. 10.1089/jir.2007.0003 17892399

[42] TIAN G, CHEN L, YU B, HUANG X, WANG J, BAHETI K et al. Association of IL-10 and TNF-α gene polymorphisms with hepatic echinococcus granulosus infection and necrosis. J Chin Physician. 2022:1504–8.

[43] Zaeemi M, Razmi GR, Mohammadi GR, Abedi V, Yaghfoori S. Evaluation of serum biochemical profile in Turkoman horses and donkeys infected with Theileria equi; 2016.

[44] Bozukluhan K, Merhan O, Büyük F, Çelebi Ö, Gökçe G. Determination of some acute phase proteins level in cattle with brucellosis. Ankara Üniversitesi Veteriner Fakültesi Dergisi. 2016;63:13–6.

[45] Ramadan RM, Wahby AM, Bakry NM, Auda HM, Mohammed FF, El-Bahy MM, Hekal SHA. Targeted pre-partum strategies to suppress Toxocara vitulorum hypobiotic larvae: reducing transmission to calves and genotypic insights into Buffalo infections. Veterinary World. 2025;18(2):329–40. 10.14202/vetworld.2025.329-340. 10.14202/vetworld.2025.329-340 PMC11963570 40182831

[46] Hassanzadeh E, Khademvatan S, Jafari B, Jafari A, Yousefi E. In vitro and in Silico scolicidal effect of sanguinarine on the hydatid cyst Protoscoleces. PLoS ONE. 2023;18:e0290947–0290947. 10.1371/journal.pone.0290947. 37878663 10.1371/journal.pone.0290947 PMC10599545

[47] Albani CM, Borgo J, Fabbri J, Pensel P, Paladini A, Beer MF, et al. Antiparasitic effects of Asteraceae species extracts on Echinococcus granulosus S.s. Evid Based Complement Alternat Med. 2022;2022:6371849. 10.1155/2022/6371849. 36193140 10.1155/2022/6371849 PMC9526667

[48] Kowalska M, Nowaczyk J, Fijałkowski Ł, Nowaczyk A. Paroxetine-Overview of the molecular mechanisms of action. Int J Mol Sci. 2021;22:1662. 10.3390/ijms22041662. 33562229 10.3390/ijms22041662 PMC7914979

[49] Weeks JC, Roberts WM, Leasure C, Suzuki BM, Robinson KJ, Currey H, et al. Sertraline, Paroxetine, and chlorpromazine are rapidly acting anthelmintic drugs capable of clinical repurposing. Sci Rep. 2018;8:975. 10.1038/s41598-017-18457-w. 29343694 10.1038/s41598-017-18457-w PMC5772060

[50] Herz M, Brehm K. Serotonin stimulates Echinococcus multilocularis larval development. Parasit Vectors. 2021;14:14. 10.1186/s13071-020-04533-0. 33407815 10.1186/s13071-020-04533-0 PMC7789706

[51] Camicia F, Vaca HR, Guarnaschelli I, Koziol U, Mortensen OV, Fontana ACK. Molecular characterization of the serotonergic transporter from the cestode Echinococcus granulosus: Pharmacology and potential role in the nervous system. Parasitol Res. 2022;121:1329–43. 10.1007/s00436-022-07466-y. 35169884 10.1007/s00436-022-07466-y PMC9487190

[52] Köhler P, Bachmann R. Intestinal tubulin as possible target for the chemotherapeutic action of Mebendazole in parasitic nematodes. Mol Biochem Parasitol. 1981;4:325–36. 10.1016/0166-6851(81)90064-5. 7335116 10.1016/0166-6851(81)90064-5

[53] Ranjan P, Kumar SP, Kari V, Jha PC. Exploration of interaction zones of β-tubulin Colchicine binding domain of helminths and binding mechanism of anthelmintics. Comput Biol Chem. 2017;68:78–91. 10.1016/j.compbiolchem.2017.02.008. 28259774 10.1016/j.compbiolchem.2017.02.008

[54] Rehan. design synthesis and biological evaluation of novel targeted anthelmintic agents. 2020. https://www.napier.ac.uk/research-and-innovation/research-search/phds/design-synthesis-and-biological-evaluation-of-novel-targeted-anthelmintic-agents. Accessed 5 Jan 2025.

[55] Hafeez MA, Sattar A, Aslam F, Imran M, Ashraf K, Zia R, Mehdi MM. Effects of ibuprofen and clopidol alone and in combination on experimentally induced coccidiosis in broiler chickens. Pakistan J Zool. 2022. 10.17582/journal.pjz/20180620100651.

[56] Hafeez MA, Sattar A, Ashraf K, Mehdi M, Rafique A, Mahmood MS et al. Effect of ibuprofen alone and inconjugation with vitamin E and selenium on experimentally induced coccidiosis in Broliers. JAPS. 2020;30.

[57] Rojo-Arreola L, Long T, Asarnow D, Suzuki BM, Singh R, Caffrey CR. Chemical and genetic validation of the Statin drug target to treat the helminth disease, schistosomiasis. PLoS ONE. 2014;9:e87594–87594. 10.1371/journal.pone.0087594. 24489942 10.1371/journal.pone.0087594 PMC3906178

[58] Canavese M, Crisanti A. Vascular endothelial growth factor (VEGF) and Lovastatin suppress the inflammatory response to plasmodium Berghei infection and protect against experimental cerebral malaria. Pathog Glob Health. 2015;109:266–74. 10.1179/2047773215Y.0000000021. 26392164 10.1179/2047773215Y.0000000021 PMC4727581

[59] Mota S, Bensalel J, Park DH, Gonzalez S, Rodriguez A, Gallego-Delgado J. Treatment reducing endothelial activation protects against experimental cerebral malaria. Pathogens. 2022;11:643. 10.3390/pathogens11060643. 35745497 10.3390/pathogens11060643 PMC9229727

[60] Nishi L, Santana PL, Evangelista FF, Beletini LF, Souza AH, Mantelo FM, et al. Rosuvastatin reduced brain parasite burden in a chronic toxoplasmosis in vivo model and influenced the neuropathological pattern of ME-49 strain. Parasitology. 2020;147:303–9. 10.1017/S0031182019001604. 31727196 10.1017/S0031182019001604 PMC10317618

[61] Sanfelice RAda, Da Silva SS, Bosqui LR, Machado LF, Miranda-Sapla MM, Panagio LA, et al. Pravastatin and Simvastatin pretreatment in combination with pyrimethamine and sulfadiazine reduces infection process of Toxoplasma gondii tachyzoites (RH Strain) in HeLa cells. Acta Parasitol. 2019;64:612–6. 10.2478/s11686-019-00076-2. 31286354 10.2478/s11686-019-00076-2

[62] Kumar GA, Roy S, Jafurulla M, Mandal C, Chattopadhyay A. Statin-induced chronic cholesterol depletion inhibits leishmania donovani infection: relevance of optimum host membrane cholesterol. Biochim Et Biophys Acta (BBA) - Biomembr. 2016;1858:2088–96. 10.1016/j.bbamem.2016.06.010. 10.1016/j.bbamem.2016.06.010 27319380

[63] Parihar SP, Hartley M-A, Hurdayal R, Guler R, Brombacher F. Topical Simvastatin as Host-Directed therapy against severity of cutaneous leishmaniasis in mice. Sci Rep. 2016;6:33458. 10.1038/srep33458. 27632901 10.1038/srep33458 PMC5025842

[64] Burgess V, Maya JD. Statin and aspirin use in parasitic infections as a potential therapeutic strategy: A narrative review. Rev Argent Microbiol. 2023;55:278–88. 10.1016/j.ram.2023.01.006. 37019801 10.1016/j.ram.2023.01.006

[65] Caglar R, Yuzbasioglu MF, Bulbuloglu E, Gul M, Ezberci F, Kale IT. In vitro effectiveness of different chemical agents on scolices of hydatid cyst. J Invest Surg. 2008;21:71–5. 10.1080/08941930701883640. 18340623 10.1080/08941930701883640

